# Genetic variations for egg quality of chickens at late laying period revealed by genome-wide association study

**DOI:** 10.1038/s41598-018-29162-7

**Published:** 2018-07-17

**Authors:** Zhuang Liu, Congjiao Sun, Yiyuan Yan, Guangqi Li, Fengying Shi, Guiqin Wu, Aiqiao Liu, Ning Yang

**Affiliations:** 1 National Engineering Laboratory for Animal Breeding and MOA Key Laboratory of Animal Genetics and Breeding, College of Animal Science and Technology, China Agricultural University, Beijing, 100193 China; 2 Beijing Engineering Research Center of Layer, Beijing, 101206 China

## Abstract

With the extension of the egg-laying cycle, the rapid decline in egg quality at late laying period has aroused great concern in the poultry industry. Herein, we performed a genome-wide association study (GWAS) to identify genomic variations associated with egg quality, employing chicken 600 K high-density SNP arrays in a population of 1078 hens at 72 and 80 weeks of age. The results indicated that a genomic region spanning from 8.95 to 9.31 Mb (~0.36 Mb) on GGA13 was significantly associated with the albumen height (AH) and the haugh unit (HU), and the two most significant SNPs accounted for 3.12 ~ 5.75% of the phenotypic variance. Two promising genes, *MSX2* and *DRD*1, were mapped to the narrow significant region, which was involved in embryonic and ovary development and found to be related to egg production, respectively. Moreover, three interesting genes, *RHOA*, *SDF4* and *TNFRSF4*, identified from three significant loci, were considered to be candidate genes for egg shell colour. Findings in our study could provide worthy theoretical basis and technological support to improve late-stage egg quality for breeders.

## Introduction

Eggs are rich in protein, fatty acids, vitamins and minerals, etc. and are considered as an excellent source of animal protein. With the improvement of laying hens’ production performance, the laying age of commercial laying hens has been extended from the original 72 weeks to 80 weeks, and some breeding companies have even extended the laying cycle to 100 weeks, proposing the breeding programme “Breeding for 500 eggs in 100 weeks”^1,2^. However, the rapid decline in egg quality at the end of the laying cycle (such as the low commercialization rate along with enlarged egg size, declined egg shell quality, increased egg broken rate (%cracks), decreased albumen height, and shortened egg storage time) has badly hindered the achievement of this goal. Therefore, improving egg quality plays an important role in realizing this programme and extending the laying cycle.

Egg quality is a comprehensive concept that encompasses both internal and external quality and includes many aspects. External quality is composed of eggshell colour, egg shape index, eggshell thickness and eggshell strength, while internal quality refers to albumen height, egg yolk colour and haugh unit. All of these are quantitative traits. With the development of molecular genetics, many studies have been carried out to reveal the genetic determination for egg quality. Microsatellite markers were first employed, and many QTLs were reported through linkage analysis^3–6^, mostly based on F2 crossed populations. Currently, there are 430 QTLs reported to be associated with egg quality in the Animal QTL database (https://www.animalgenome.org/cgi-bin/QTLdb/GG/index)^7^. Although many studies identified QTLs, they had wide confidence intervals for position and were rarely replicated^8,9^. A new era began with the subsequent advance in SNP chip and sequencing technology, and the genome wide association study (GWAS) has become one of the most effective methods to detect genetic variations in livestock. Liu *et al*. carried out the first GWAS with the Illumina 60 K SNP array^10^ to uncover the genetic associations with egg quality traits in chickens^11^. Wolc *et al*. and Sun *et al*. subsequently reported many genes related to egg quality that were identified by genome-wide association studies^12,13^. Despite all this, the genetic improvement of egg quality is slow because of relatively low heritabilities and intensively artificial selection on egg production.

To our knowledge, little research reported the genetic analysis for egg quality in the late laying period of chickens. In the present study, we employed the commercial chicken 600 K SNP chip to detect the genetic variations associated with egg quality in a population of 1078 hens at 72 and 80 weeks of age by genome-wide association analysis (GWAS) to provide a theoretical basis and technological support for improving late-stage egg quality.

## Results

### Phenotypic statistics and estimation of genetic parameter

The descriptive statistics for eggshell colour (ESC), egg shape index (ESI), eggshell thickness (EST), eggshell strength (ESS), albumen height (AH), yolk colour (YC) and haugh unit (HU) at 2 age points were presented in Table 1. With the extension of the laying period, the phenotypic values of ESS, AH and HU at 80 weeks of age decreased compared to the values at 72 weeks of age. Both eggshell strength and albumen height at the two points had higher phenotypic variation (19~30%) than the other traits. The pedigree-based hereditability were high for ESC, ESI, AH and HU (0.32~0.46) and moderate for EST, ESS and YC (0.14~0.28).

**Table 1:** Descriptive statistics for egg quality traits.

Traits^a^	N	Mean	SD	CV (%)	Min	Max	h^2^ (SE)
ESC72	940	62.36	4.22	6.78	34.18	79.20	0.37 (0.09)
ESI72	904	1.29	0.05	4.15	1.04	1.52	0.32 (0.08)
EST72	914	0.28	0.03	11.01	0.21	0.39	0.14 (0.07)
ESS72	900	3.17	0.94	29.79	0.59	5.37	0.27 (0.08)
AH72	902	6.09	1.14	18.66	2.20	10.30	0.36 (0.09)
YC72	902	9.29	0.42	4.53	7.70	11.30	0.14 (0.07)
HU72	902	76.25	9.55	12.53	21.90	99.10	0.40 (0.09)
ESC80	831	62.42	4.34	6.95	50.74	83.27	0.42 (0.09)
ESI80	816	1.30	0.05	4.03	1.14	1.49	0.46 (0.10)
EST80	808	0.28	0.03	10.80	0.21	0.39	0.28 (0.09)
ESS80	811	2.99	0.89	29.90	0.39	5.09	0.25 (0.08)
AH80	812	5.36	1.25	23.33	2.20	9.70	0.38 (0.10)
YC80	812	9.32	0.44	4.77	7.50	11.30	0.35 (0.09)
HU80	812	69.68	11.50	16.51	31.50	97.20	0.43 (0.10)

N: number of samples, ESC: eggshell color, ESI: egg shape index, EST: eggshell thickness, ESS: eggshell strength, AH: albumen height, YC: yolk color, HU: haugh unit, SD: standard deviation, CV: coefficient of variance, h^2^ (SE):pedigree-based hereditability (standard error).

Traits^a^: ESC, ESI, EST, ESS, AH, YC, HU at 72 and 80 weeks of age.

The estimates of SNP-based heritability and genetic or phenotypic correlations among egg quality traits at the two points are shown in Table 2 and Supplementary Table S1 (at 80 weeks of age). The estimates of SNP-based heritability were lower than those for pedigree-based heritability for all traits except EST (0.20 vs 0.14). Regardless of genetic relation or phenotypic correlation, HU has a strong correlation with AH (0.97 ± 0.02, 0.94), while ESC has very weak correlation with the other traits.

**Table 2:** Estimation of genetic parameters for egg quality traits.

Traits^a^	ESC72	ESI72	EST72	ESS72	AH72	YC72	HU72
ESC72	**0.34 (0.06)**	0.03 (0.15)	0.09 (0.18)	−0.06 (0.17)	0.06 (0.19)	−0.04 (0.21)	0.16 (0.18)
ESI72	0.02	**0.30 (0.06)**	−0.05 (0.18)	−0.00 (0.17)	−0.41 (0.17)	0.04 (0.20)	−0.40 (0.17)
EST72	0.00	−0.06	**0.20 (0.06)**	0.80 (0.12)	−0.27 (0.21)	0.49 (0.24)	−0.31 (0.21)
ESS72	−0.07	−0.07	0.47	**0.23 (0.06)**	0.00 (0.20)	0.53 (0.22)	−0.02 (0.20)
AH72	−0.06	−0.16	−0.03	0.01	**0.19 (0.06)**	−0.35 (0.24)	0.97 (0.02)
YC72	−0.09	0.03	0.06	0.06	−0.06	**0.14 (0.05)**	−0.34 (0.24)
HU72	−0.04	−0.15	−0.04	0.01	0.94	−0.10	**0.19** (**0.06)**

Diagonal: heritability estimation (bold is SNP-based), Upper triangle: genetic correlations, Lower triangle: phenotypic correlations. Standard error are in parenthese.

Traits^a^: ESC, ESI, EST, ESS, AH, YC, HU at 72 weeks of age.

### Genome-wide association study (GWAS)

**Figure 1:**
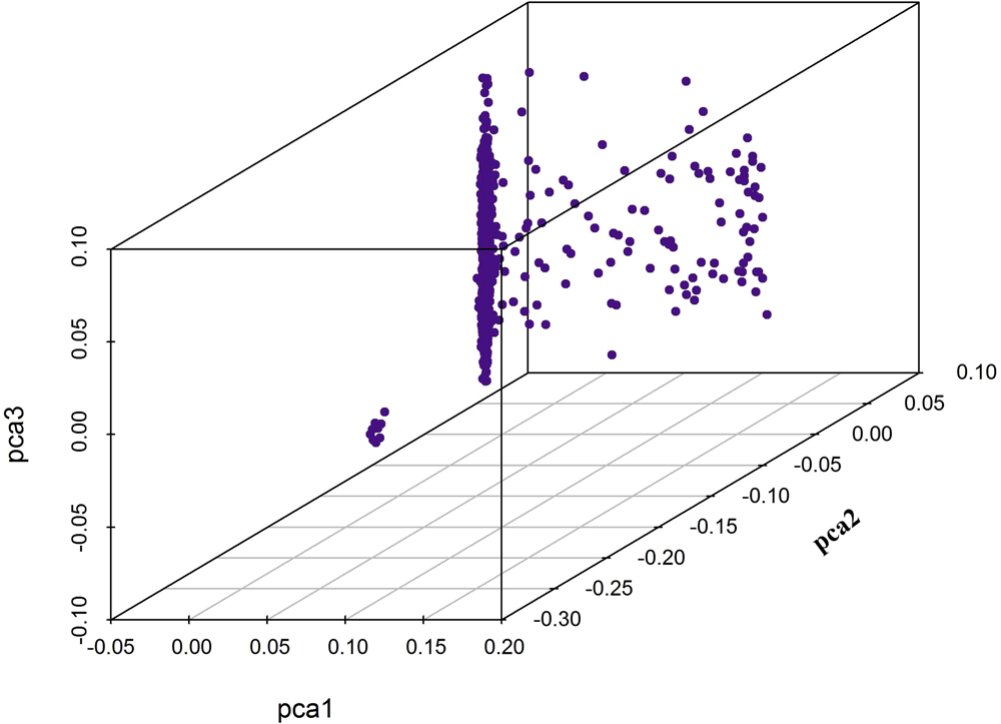
Principal component plot (3D) of chickens using SNP markers. Purple points represent different individuals.

The analysis of the population structure was shown in Fig. 1. As shown in the 3D plot, the samples had a slight population stratification. Therefore, we treated the first five principal components as covariates and included them in the linear mixed model of GWAS as fixed effects to adjust for the population structure effects.

**Figure 2:**
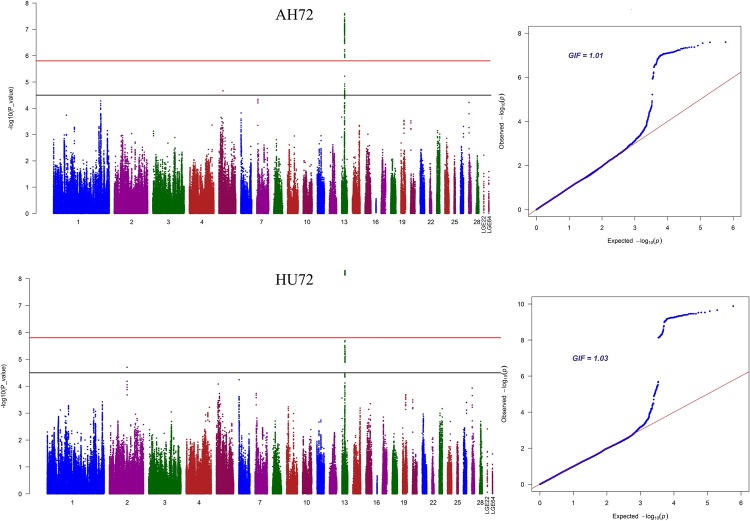
Manhattan plots and Q-Q plots of genome-wide association studies for AH and HU. Each dot represents an SNP in the dataset. The horizontal red and black lines indicate the genome-wide significant threshold (1.58e–6) and genome-wide suggestive significant threshold (3.17e–5), respectively. The Manhattan plots indicate −log_10_ (observed P-values) for genome-wide SNPs (y-axis) against their corresponding position on each chromosome (x-axis), while the Q-Q plots show the expected -log_10_-transformed P-values vs. the observed -log_10_-transformed P-values. AH and HU denote albumen height and haugh unit, respectively. GIF represents genomic inflation factor.

**Figure 3:**
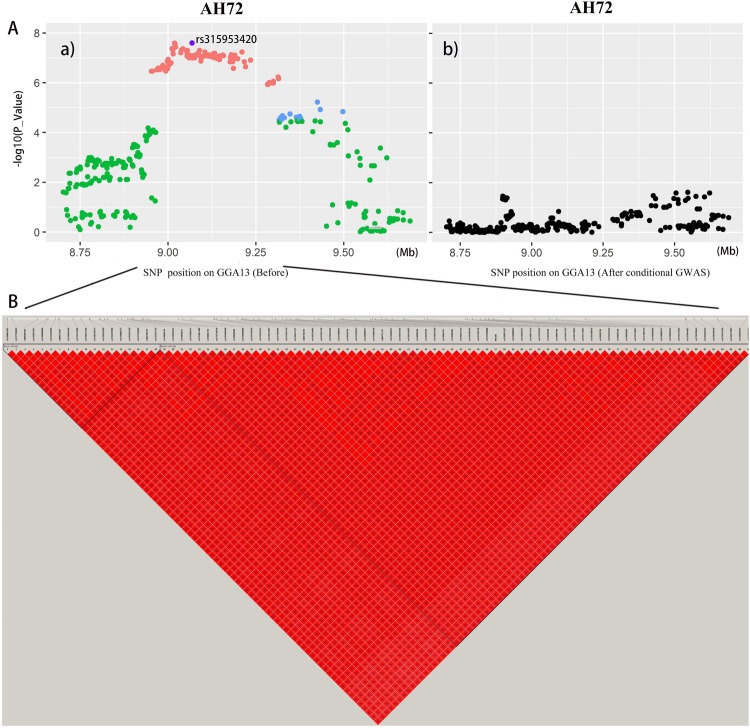
Conditional GWAS of AH (**A**) and LD analysis of loci in the significant region (**B**). Conditional association analyses were carried out by fitting the most significant SNP rs315953420 as a covariate. The strong LD block is defined as D’ ≥ 0.8.

The Manhattan and Quantile-Quantile (QQ) plots of seventeen separate GWASs using the univariate model or the bivariate model for egg quality traits are shown in Fig. 2 and Supplementary Fig. S1. The univariate analyses revealed 99 and 86 significant SNPs (Supplementary Table S2) associated with AH and HU (Fig. 2), respectively. Combined with the bivariate model analyses, all these significant SNPs were identified as being located in an ~0.36 Mb region spanning from 8.95 to 9.31 Mb on GGA13 because of the strong genetic correlations. We calculated the genomic control inflation factors (λ) of AH and HU, which ideally equal 1. In fact, they were slightly greater than 1 (1.01, 1.03), which reflected the slight population stratification. These results were also consistent with the previous principal component analyses (PCA). Linkage disequilibrium (LD) analysis uncovered SNPs in GGA13 from 8.95 to 9.31 Mb that showed strong LD (Fig. 3B, Supplementary Fig. S2). To find the independent SNPs, stepwise conditional analyses were carried out. The locus rs315953420 significantly associated with AH and HU at the two periods was subsequently added to the model to examine the independent associations (Fig. 3Aa). The level of all significant or suggestive loci around the rs315953420 SNP decreased below the genome-wide suggestive threshold after treating the genotype of the locus as a covariate in the conditional GWAS (Fig. 3Ab)). Another SNP in the significant region, rs15695238, was identified as being associated with AH and HU.

**Figure 4:**
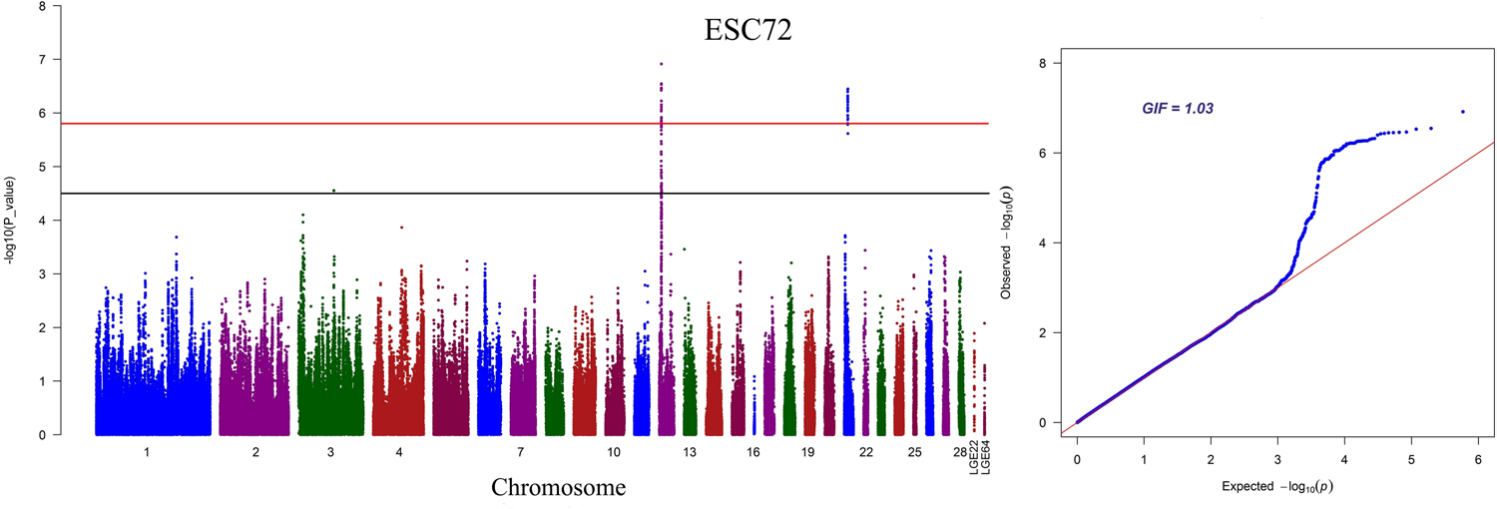
Manhattan plots and Q-Q plots of genome-wide association study for ESC at 72 weeks of age. The horizontal red and black lines represent the genome-wide significant threshold (1.58e–6) and genome-wide suggestive significant threshold (3.17e–5), respectively. ESC denotes eggshell colour. GIF indicates genomic inflation factor.

**Figure 5:**
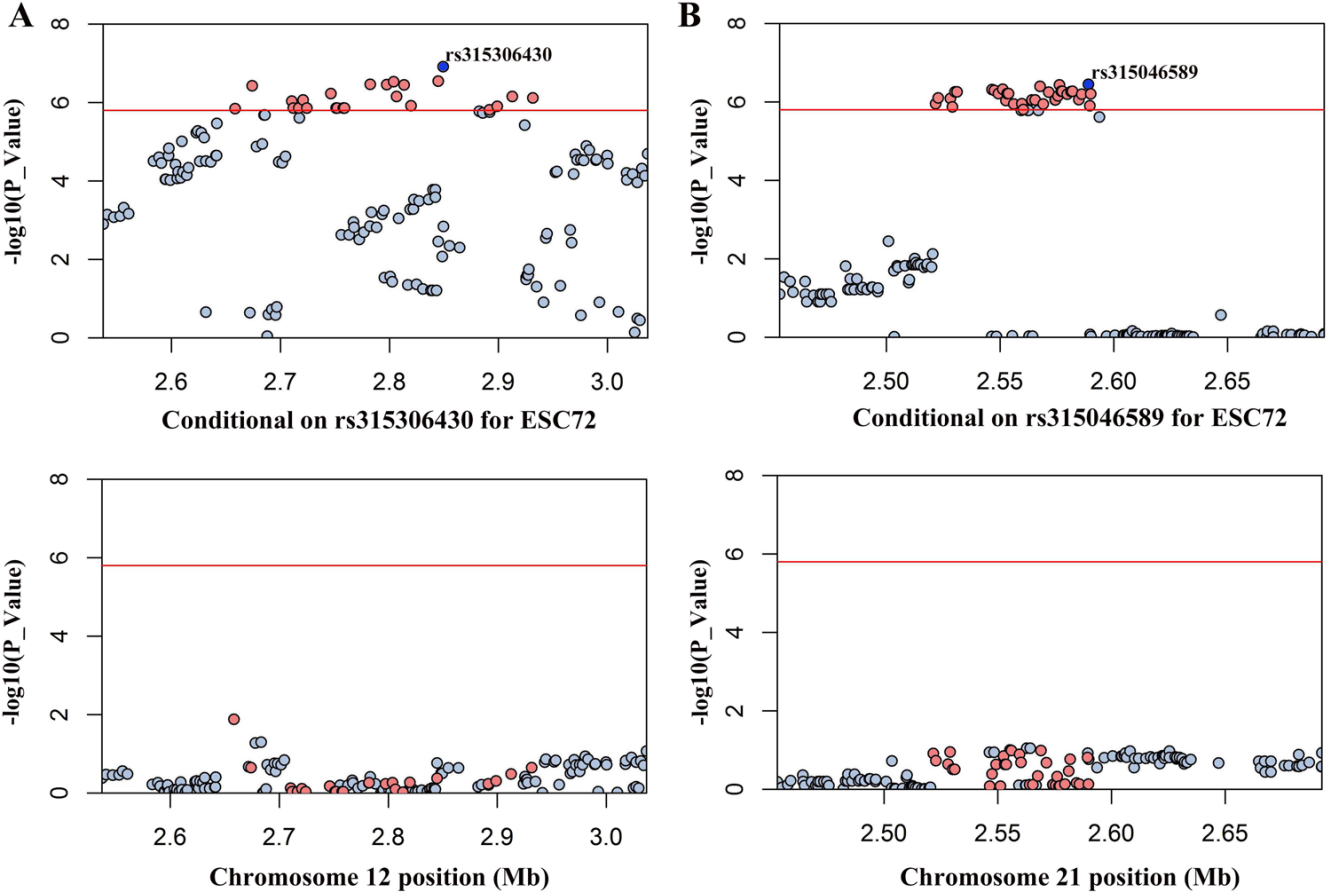
Regional association plots of two loci associated with ESC72. Each plot shows the −log_10_ (observed P-values) against their chromosomal positions. The horizontal red line represents the genome-wide significance level (1.58e–6). (**A)** Depicts the results for ESC72 before (upper) and after (lower) the inclusion of rs315306430. (**B)** Depicts the results for ESC72 before (upper) and after (lower) condition on rs315046589.

As shown in Fig. 4, there were 24 and 37 hits significantly associated with ESC on GGA12 and GGA21, respectively (Supplementary Table S2). LD analysis showed that 24 SNPs on GGA12 were clustered into two neighbour blocks, Block 1 (42 kb) and Block 2 (191 kb) (Supplementary Fig. S2). All the significant SNPs on GGA21 were in a strong LD block (Supplementary Fig. S2). Then, we separately added the two most significant SNPs (rs315306430 and rs315046589) into the model of conditional analysis. The significant difference in levels is displayed in Fig. 5 (upper panel was before the addition of the SNPs, while the lower panel was after). In addition, four SNPs (rs14283106, rs317521562, rs13603074 and rs16179081) were identified by the bivariate analysis as affecting phenotypes at the two time points. Unfortunately, no genome-wide significant locus was screened for ESI, EST, ESS or YC.

### SNP annotation and promising genes associated with egg quality

The annotation of significant SNPs using the Variant Effect Predictor (VEP) tool supplied by Ensembl could help us identify promising genes associated with egg quality traits. The detailed information about the genes was summarized in Table 3. For AH and HU, two significant loci in an ~0.36 Mb (8.95 to 9.31 Mb) significant region on GGA13 were detected to have substitutions of C to A and T to C, respectively. The SNP rs315953420 was located 60 Kb upstream of msh homeobox 2 (*MSX2*), while the SNP rs15695238 was located 170 Kb downstream of dopamine receptor D1 (*DRD*1). Egg shell colour has high heritability and is affected by polygenes. Five candidate genes, namely, ras homologue family member A (*ROHA*), stromal cell derived factor 4 (*SDF4*), TNF receptor superfamily member 4 (*TNFRSF4*), tubulin tyrosine ligase-like 10 (*TTLL1*0), and *LOC419425*, and one microRNA (*MIR429*) were found close to the significant SNPs associated with ESC (Table 3).

**Table 3:** The annotation of significant SNPs associated with AH, HU and ESC.

Traits	GGA^b^	Tag SNP	Position	Alt/Ref allele	Location (Kb)	Gene symbol
AH72/80^a^	13	rs315953420	9068564	C/A	upstream_60	MSX2
HU72/80^a^	13	rs15695238	9186255	T/C	downstream_170	DRD1
ESC72	12	rs315306430	2849211	T/C	upstream_4.73	RHOA
ESC72	21	rs315046589	2588765	C/T	Downstream_6.66	MIR429
ESC72/80^a^	21	rs14283106	2546399	G/A	downstream_0.84	SDF4
ESC72/80^a^	21	rs317521562	2551092	A/G	intron	TNFRSF4
ESC72/80^a^	21	rs13603074	2576858	T/C	intron	TTLL10
ESC72/80^a^	21	rs16179081	2575378	A/G	intron	LOC419425

^a^Indicating SNPs reached genome-wide significance in univariate and bivariate GWAS. AH: albumen height, HU: haugh unit, ESC: eggshell color.

^b^Chicken chromosome.

### SNP contribution to phenotypic variation

**Figure 6:**
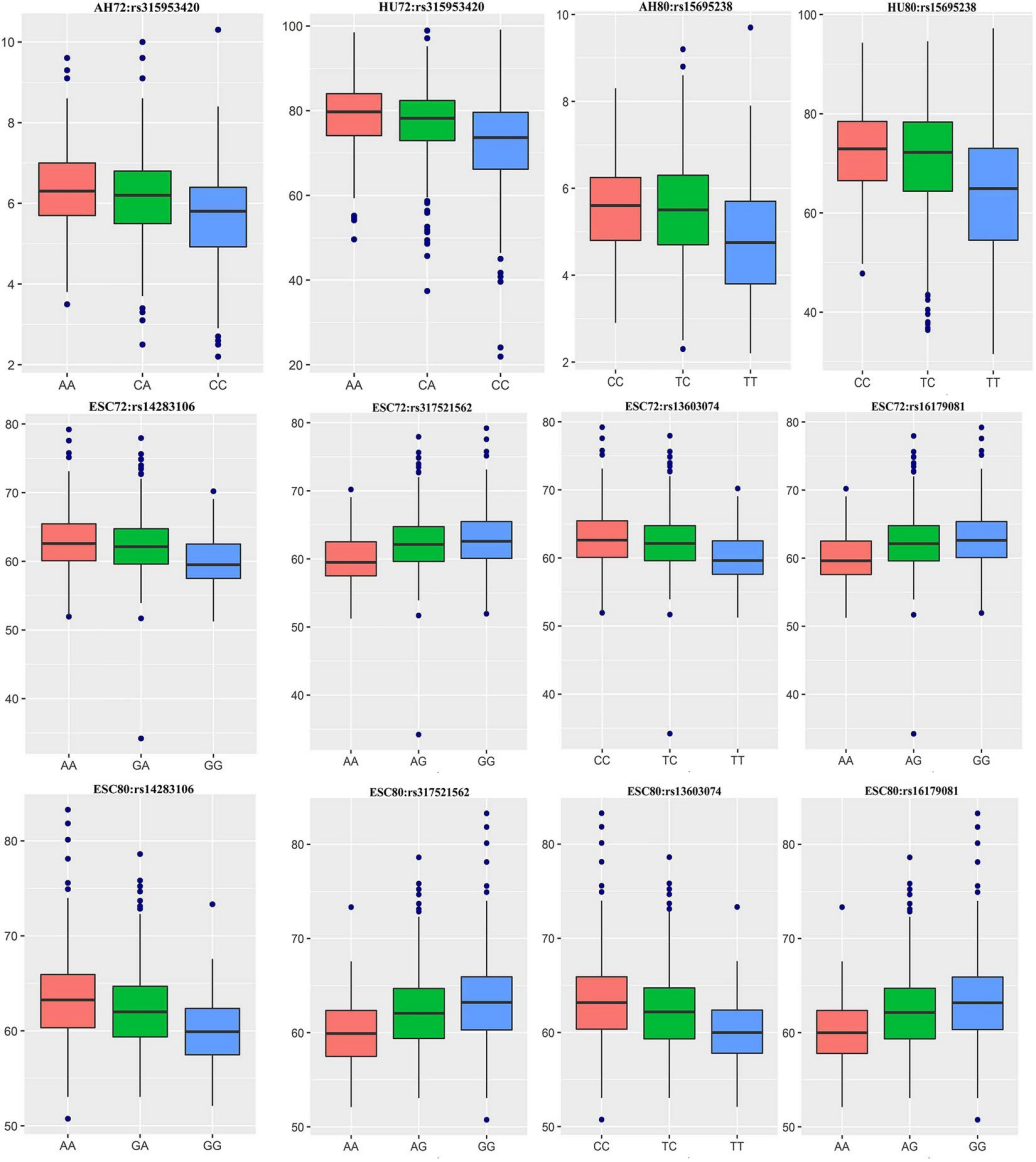
Boxplots of SNP effects on AH, HU and ESC at 72 and 80 weeks of age. The figure shows the genotypes of six representative SNPs (x-axis) versus the phenotypic values of the corresponding traits (y-axis). AH, HU and ESC represent albumen height, haugh unit and eggshell colour, respectively.

As shown in Table 4, we extracted six meaningful loci which reached genome-wide significance in the univariate and bivariate GWAS for further analysis: rs315953420, rs15695238, rs14283106, rs317521562, rs13603074 and rs16179081 in *MSX2*, *DRD1*, *SDF4*, *TNFRSF4*, *TTLL10* and *LOC419425*, respectively. The effect allele frequency (EAF) ranged from 0.356 to 0.465, which meant that these SNPs were separate. For AH and HU, the phenotypic variance explained by two significant SNPs ranged from 3.12 to 5.75%. The substitution of one copy of EA at the rs31594320 site caused the highest decrease (0.339 SD/allele) in haugh unit at 72 weeks of age. The remaining four SNPs accounted for 3.28~4.15% of the variance in ESC. In addition, we compared actual phenotypic differences among the three genotypes of the above SNPs (Fig. 6). The results revealed that the phenotypes corresponding to the different genotypes displayed significant segregation. The homozygotes of the effect allele or the alternative allele resulted in the lowest and highest individual phenotypes, respectively, while the heterozygotes were intermediate.

**Table 4:** Contribution of significant SNPs to AH, HU and ESC.

SNP		rs315953420	rs15695238	rs14283106	rs317521562	rs13603074	rs16179081
Chromosome		13	13	21	21	21	21
Position (bp)		9068564	9186255	2546399	2551092	2576858	2575378
EA/AA		C/A	T/C	G/A	A/G	T/C	A/G
EAF		0.465	0.464	0.359	0.359	0.364	0.356
AH72	Beta (SE)^a^	−0.299 (0.053)	−0.275 (0.053)	—	—	—	—
	CPV (%)	4.33	3.70	—	—	—	—
AH80	Beta (SE)	−0.257 (0.057)	−0.266 (0.057)	—	—	—	—
	CPV (%)	3.12	3.42	—	—	—	—
HU72	Beta (SE)	−0.339 (0.052)	−0.317 (0.052)	—	—	—	—
	CPV (%)	5.75	5.08	—	—	—	—
HU80	Beta (SE)	−0.273 (0.057)	−0.279 (0.056)	—	—	—	—
	CPV (%)	3.64	3.83	—	—	—	—
ESC72	Beta (SE)	—	—	−0.276 (0.054)	−0.276 (0.054)	−0.279 (0.055)	−0.271 (0.054)
	CPV (%)	—	—	3.42	3.44	3.39	3.28
ESC80	Beta (SE)	—	—	−0.297 (0.057)	−0.291 (0.058)	−0.293 (0.059)	−0.285 (0.058)
	CPV (%)	—	—	4.15	4.00	3.93	3.79

Abbreviations: EA/AA: effect allele (minor allele)/alternative allele (major allele), EAF: effect allele frequency, AH72, AH80, HU72, HU80, ESC72, ESC80: albumen height, haugh unit and eggshell color of 72, 80 weeks age;

^a^Estimated allelic substitution effect per copy of the effect allele (EA) based on an inverse-normal transformed scale under an additive model, expressed in SD unit/allele; SE: standard error of the beta;

CPV: contribution to phenotypic variance (%).

## Discussion

Egg quality is an important economic trait. With the extension of the laying cycle, the decline in egg quality during the late laying period, such as the increased %cracks and the large variation in eggshell colour, has aroused great concern^2^. The present research was designed to uncover the key genes that affect egg quality using egg quality data from chickens with laying ages of 72 and 80 weeks. This study is the first to conduct GWASs of egg quality at the late laying period, employing the chicken 600 K high-density SNP chip.

The population employed in this study was a purebred brown egg-type chicken line, rather than a cross between two or more distant populations. This decreased the power to detect QTLs for egg quality traits that differ between the crossed populations^14^, but this was compensated for by the numerous genotypes and phenotypes of the individuals and the effective methods used^15^. The phenotypic values of AH and HU at 80 weeks of age decreased compared to those at 72 weeks of age. Previous research has indicated that the rapid reduction in egg quality during the late laying period could lead to a decrease in hatchability^2^. The pedigree-based heritability estimates of ESC, AH and YC were slightly lower than those in the Hy-line resource population, which were evaluated at 42 and 46 weeks of age^12^. The estimates of ESS and EST were similar to those in the F_2_ population at 72 weeks of age^13^. The genetic and phenotypic correlation of all egg quality traits was significantly reduced compared with the correlations found in previous studies^16^ because of the larger coefficient of variance at late laying period. Moreover, the pedigree-based heritability estimates were larger than the SNP-based estimates. This may be caused by “missing heritability”^17^ which refers to the fact that the eligible SNPs in the Affymetrix 600 K SNP chip do not represent the complete genomic variation in chickens.

Genome-wide association analysis of egg quality traits were carried out separately. A significant genomic region of 0.36 Mb on chromosome 13 (GGA13) that harboured 85 unique SNPs (Supplementary Table S2) was identified as being associated with albumen height (AH) and haugh unit (HU). The results of the conditional GWAS and the linkage disequilibrium (LD) analysis revealed that the SNPs were closely linked together in this region. As listed in the chicken QTL database (https://www.animalgenome.org/cgi-bin/QTLdb/GG/index), a total of 67 QTL_IDs were reported for AH and HU. For example, four putative QTL regions were detected on GGA 1, 9, 13 and 23 that were associated with albumen height^11^, one was detected in the centre of GGA 7, identified by its proximity to a candidate gene^18^, and 5 regions were reported on GGA2, 3, 4, 9 and Z^12^. The published QTL regions on GGA13 were close to the genomic region in our study. By annotating the significant SNPs, two promising genes (*MSX2* and *DRD1*) near this region were found to be associated with AH and HU. The *MSX2* gene is a member of the msh homeobox family and is expressed in many embryonic tissues. In the developing chick, *MSX2* is expressed in the apical ectodermal ridge and the ectoderm of the genital tubercle, and it plays a crucial role in the growth and patterning of the limb mesoderm^19^. In addition, the expression of *MSX2* could influence the function of TNF-α (tumour necrosis factor-α), which is known to suppress adipocyte differentiation and to activate the Wnt/β-catenin pathway^20,21^. The Wnt signalling pathway regulates crucial aspects of cell fate determination, cell migration, and cell polarity during embryonic and ovary development^22^. In addition, a previous proteomic study reported that, in eggs, a portion of the functions of the proteins were associated with embryonic development^13^. We know that the egg proteins are secreted by the magnum during the egg-laying process. Therefore, we believe that *MSX2* gene has an indirect effect on AH and HU during the process of protein formation. Another gene, *DRD1*, belongs to the D1-like type of dopamine receptor. In birds, dopamine has been revealed to be involved in both stimulating and inhibiting prolactin (PRL) secretion, which has been demonstrated to play a crucial role in the onset and maintenance of incubation behaviour^23–25^. Dopamine stimulates PRL secretion by activating *DRD1* at the hypothalamus level and inhibits PRL secretion through *DRD2* at the pituitary level^26–29^. All these studies have indicated that the dopamine receptor participates in the system of regulating avian reproductive behaviour. Like other D1-like members, the chicken *DRD1* is an intronless gene and belongs to the rhodopsin family. Recently, Schnell *et al*. and Chaiseha *et al*. demonstrated that the *DRD1* gene is widely expressed in the hypothalamus and pituitary and that the expression is associated with the functioning of the reproductive system in turkeys^30,31^. Moreover, previous findings have revealed that the *DRD1* gene and its haplotypes are associated with some egg production traits in chickens^32^. We suggest that *DRD1* could be treated as a candidate gene related to egg quality in further analyses.

There is extensive scientific literature regarding eggshell colour, and some studies have concluded that the green colour of the eggshell is controlled by a single dominant gene (An EAV-HP Insertion in 5′ Flanking Region of *SLCO1B3* Causes Blue Eggshell in the Chicken)^33,34^, but there still exists much uncertainty about the molecular mechanism underlying brown eggshell colour, even though it has been reported to be controlled by multiple minor genes^5,12,35,36^. Li *et al*. used quantitative PCR to determine the expression levels of 8 genes encoding enzymes in the liver and shell gland in a Rhode Island Red pure line. The interesting gene *ABCG2* might facilitate the accumulation of protoporphyrin IX, which is the main pigment resulting in the brown coloration of eggshell^36^. In addition, Wolc *et al*. found two genomic regions located on GGA4 and GGA12 that affect the early and late eggshell colour, respectively, in a pureline Hy-line population^12^. In the present study, one gene (*RHOA*) on GGA12 was related to ESC at 72 weeks of age, and four genes, namely, *SDF4*, *TNFRSF4*, *TTLL10* and *LOC419425*, were identified by GWAS as being associated with egg shell colour (ESC) at the two periods. The SNP rs315306430 located 4.73 Kb upstream of *RHOA* overlapped with a previously identified QTL^12^. *RHOA* is a small GTPase and belongs to the ras homologue (Rho) family. The Rho family of small GTPases are molecular switches that control a wide variety of cell functions, including cytoskeletal reorganization, cell motility, and gene expression^37^. In addition, it has been revealed that the *RHOA* signalling system plays a role in the modulation of actin stress fibres and chondrogenesis^38^. *SDF4* is the stromal cell derived factor 4, and its human orthologue is known as *Cab45*. A 45-kDa Ca^2+^-binding protein, *Cab45* is important because it can regulate cell migration through various molecular mechanisms^39^. Another gene, *TNFRSF4*, encodes proteins and can be used to specifically modulate the expression of other genes that directly stimulate effector T-cell activity^40^. All the above three genes are associated with body growth and phylogenesis. Recently, a very interesting study reported that eggshell pigment deposition and eggshell coloration were strongly and positively correlated with phylogenesis^41^. Therefore, we conclude that *RHOA*, *SDF4*, and *TNFRSF4* affect eggshell colour through the process of phylogenesis. Unfortunately, little information about *TTLL10* and *LOC419425* could be found in the NCBI GenBank.

In summary, we carried out univariate, bivariate and conditional GWASs for egg quality at late laying period, employing the chicken 600 K high density SNP array. A genomic region spanning from 8.95 to 9.31 Mb (~0.36 Mb) in GGA13 was detected to be significantly associated with albumen height and haugh unit. Two promising genes, *MSX2* and *DRD1*, were mapped to that narrow region. Furthermore, three interesting genes, *RHOA*, *SDF4* and *TNFRSF4*, identified from three significant loci, were considered candidate genes related to egg shell colour. However, further functional validation needs to be performed in chickens. Findings in our study could provide worthy theoretical basis and technological support to improve late-stage egg quality for breeders.

## Materials and Methods

### Ethics statements

All the blood sample collections were performed in accordance with the Guidelines for Experimental Animals established by the Ministry of Agriculture of China (Beijing, China). The whole study was approved by the Animal Welfare Committee of China Agricultural University (Permit Number: SYXK 2007-0023).

### Resource Population

An 11^th^ generation population of Rhode Island Red chickens from the Beijing Huadu Yukou Poultry Breeding Co., Ltd. were the experimental animals used in this study. This pure line has been selected for egg production over many generations. Thus, a total of 1,078 hens with accurate pedigrees were chosen for SNP genotyping. The hens were housed in individual cages in the same area with free access to feed and water.

### Phenotypic measurements and evaluation of heritability

Egg quality traits including eggshell colour (ESC), egg shape index (ESI), eggshell thickness (EST), eggshell strength (ESS), albumen height (AH), yolk colour (YC) and haugh unit (HU) were measured at 72 and 80 weeks of age. Due to the late laying period, we collected fresh eggs for three successive days to ensure one egg per hen. ESC was measured with a CM-2600D reflectometer (Konica Minolta, Tokyo, Japan) using the three following parameters: L* represents lightness, a* measures the balance of red and green, and b* describes hue of blue-yellow scale. Here, we only used the L* as the eggshell colour phenotypic value. The long and short diameters of each egg were measured with a Vernier calliper, and the egg shape index was calculated as the ratio of the long and short diameters. The eggshell strength (pole to pole) of each egg was measured vertically using an EFG-0502 gauge (Robotmation, Tokyo, Japan). Then, we broke the eggs to collect the internal contents, and the AH, YC and HU were measured with the EMT-5200 multi-functional egg analyser (Robotmation, Tokyo, Japan). And the HU is also a calculation from AH with egg weight. Finally, we cleaned the eggshell membrane and measured the EST of each egg with an eggshell thickness gauge (FHK, Tokyo, Japan). Descriptive statistics of all phenotypic records were handled with R version 3.3.1 software (https://www.r-project.org/).

Pedigree-based hereditability for ESC, ESI, EST, ESS, AH, YC and HU in the two periods was calculated with the average information restricted maximum likelihood (AI-REML) method supplied by the DMU v6.0 software^42^. The multi-traits general animal model was adopted in our analysis as follows:

1 $${\bf{y}}={\bf{1}}\,{\boldsymbol{\mu }}+{\bf{Za}}+{\bf{e}}$$

where y is the phenotypic value, **1** is an n x 1 vector of all 1’s, **µ** is the population mean (fixed effect), **Z** is an incidence matrix for random additive effects, **a** and **e** are the additive effect and random residual, respectively.

### Genotyping, quality control and imputation

Genomic DNA was extracted from whole blood samples using standard phenol/chloroform methods and the 1078 qualified hens were genotyped with the Affymetrix 600 K chicken SNP chip (Affymetrix, Inc. Santa Clara, CA, USA). From a preliminary set of 580,961 SNPs^43^, 6,550 SNPs with unknown physical position and 43 markers with repeated genomic coordinates were excluded. The genotype calling and quality control were carried out by the Affymetrix Power Tools v1.19.0 (APT) software, following the pipeline of the Axiom Genotyping Solution. Only individuals with dish quality control (DQC) >0.82 and call rate >97% were included in the downstream analyses. A package of ps-metrics supplied by the APT software was run to calculate the SNP quality, and the lower quality SNPs were filtered out using an R script. After the above QC steps, 1063 individuals and 517,856 SNPs remained. We also discarded SNPs on the sex chromosomes because of the low detection power for associations between phenotypes and sex chromosome genotypes. Moreover, PLINK v1.90 software was used for further quality control (minor allele frequencies (MAF) >0.01, Hardy Weinberg equilibrium (HWE) <1e–6)^44^. The remaining SNPs were used to impute some missing genotypes with the Beagle v4.0 procedure^45^. Finally, 1063 individuals and 294,705 SNPs distributed among 28 autosomes and two linkage groups (Table 5) were eligible for the subsequent genome-wide analyses.

**Table 5:** The physical map of SNP markers after quality control in the genome.

CHROM	Map Distance (Kb)^a^	No. SNPs	Density (kb/SNP)	CHROM	Map Distance (Kb)	No.SNPs	Density (kb/SNP)
1	195273.4	56347	3.5	16	496.6	234	2.1
2	148805.6	33828	4.4	17	10451.6	4729	2.2
3	110446.7	31950	3.5	18	11200.7	5298	2.1
4	90214.2	24890	3.6	19	9980.4	4705	2.1
5	59540.2	17108	3.5	20	14299.5	4632	3.1
6	34902.1	11103	3.1	21	6789.1	4602	1.5
7	36212.8	12169	3.0	22	4073.4	2141	1.9
8	28733.1	8945	3.2	23	5712.2	3289	1.7
9	23431.7	10784	2.2	24	6319.2	4262	1.5
10	19879.9	9183	2.2	25	2190.3	1391	1.6
11	19393.5	7310	2.7	26	5310.7	3287	1.6
12	19864.8	7352	2.7	27	5197.5	2761	1.9
13	17759.8	5848	3.0	28	4742.5	2664	1.8
14	15157.4	7792	1.9	LGE22^b^	743.5	35	21.2
15	12640.8	5981	2.1	LGE64	962.6	85	11.3
Total	920725.9	294705					

Map Distance (Kb)^a^ was based on the position of the last marker in the genome. (Gallus gallus 4.0);

LGE22^b^: linkage group LGE22C19W28_E50C23.

### Population structure and association analysis

Prior to the genome-wide association study (GWAS), a principal component analysis (PCA) supplied by the PLINK package was conducted to evaluate the population stratification. We pruned all SNPs to obtain independent SNPs via the option of -indep-pairwise 25 5 0.2. In addition, the kinship matrix was built through the independent SNP markers. The principal components were calculated from the linear combination of markers by the eigenvectors of the kinship matrix, which were treated as covariates and included in model of GWAS as fixed effects to control the population structure effects. We adjusted the P-value threshold of genome-wide significance using the simpleM package^46^, considering the over-conservation of the Bonferroni method. After the simpleM test, a total of 31,589 effective independent tests were obtained. Then, the genome-wide significance and suggestive significance were calculated as 1.58e–6 (0.05/31,589) and 3.17e–5 (1.00/31,589), respectively.

The univariate GWAS was first implemented using a linear mixed model to account for the associations between each trait and the effective SNPs, which was supplied by GEMMA software^47^. The statistical model was as follows:

2 $${\bf{y}}={\rm{W}}{\boldsymbol{\alpha }}+{\rm{x}}{\boldsymbol{\beta }}+{\bf{u}}+{\boldsymbol{\varepsilon }}$$

where y is the phenotypic values of n individuals in the population of interest; W is a matrix of covariates (fixed effects: top five principal components and a column of 1 s) controlling for population structure; **α** is a vector of corresponding effects that compose the intercept; **x** is the marker genotypes; **β** is the corresponding marker’s effect; **u** is a vector of random polygenic effects with a covariance structure as **u**~N (0, **K**Vg), where **K** represents a known n x n genetic relatedness matrix derived from SNP markers and Vg is the polygenic additive variance; and **ε** is vector of random residuals. The Wald statistical test was applied to test the alternative hypothesis *H*_*1*_:***β ≠*** **0** against the null hypothesis *H*_*0*_: ***β*** = ***0*** for each SNP, as F_wald_ = ${\hat{\beta }}^{2}/Var(\hat{\beta })$.

The Manhattan and quantile-quantile (Q-Q) plots were generated for each trait using the “gap” (https://cran.r-project.org/web/packages/gap/) and “qqman” (https://cran.r-project.org/web/packages/qqman/) packages in R software, which described the transformed −log_10_ of the observed P-values against the marker locations on the genome or the expected −log_10_ (P-values). Moreover, the genomic inflation factor (λ) was calculated to judge the extent of false positive signals by the “GenABEL” package in R^48^.

Furthermore, we carried out a bivariate association analysis to directly account for the effects of the genetic variants on the dynamic egg quality traits along with the two late laying periods. The mixed model was also supplied by the GEMMA software^47^.

### Linkage disequilibrium (LD) analysis and gene identification

We performed linkage disequilibrium (LD) analysis in order to characterize causal SNPs in a strong LD region where many significant SNPs were identified by the solid spine algorithm in Haploview version 4.2 as being clustered^49^. In addition, we added the genotypes of the most significant SNPs (coded as 0, 1 or 2) as covariates into the univariate and multivariate models to elucidate independent signals in the step-wise conditional analysis. The information of significant SNPs were obtained with the annotation of Gallus-gallus 4.0, and candidate genes within 500 kb regions flanking the associated SNPs were identified using Variant Effect Predictor (VEP)^50^ supplied by Ensembl (http://www.ensembl.org).

### Estimation of genetic parameters and contribution to phenotypic variance (CPV)

All SNP-based heritability (h^2^_snp_)^51^ and pair-wise genetic correlations of egg quality traits were calculated using the restricted maximum likelihood (REML) method with GCTA v1.24 software^52^. A matrix of genetic relationships was constructed from all eligible SNPs on autosomes and two linkage groups. We then calculated the phenotypic variance contribution of those genome-wide significant SNPs based on the genetic matrix.

## Electronic supplementary material

Supplementary Dataset
