# Supplementary material for: Genetic variations for egg quality of chickens at late laying period revealed by genome-wide association study
Source: Sci Rep. 2018 Jul 17;8:10832. doi: 10.1038/s41598-018-29162-7 (PMC6050282; doi:10.1038/s41598-018-29162-7)
Supplement: Supplementary file 1 — Supplementary Dataset [file 41598_2018_29162_MOESM1_ESM.pdf]

Genetic variations for egg quality of chickens at late laying period revealed by genome-wide association study

Zhuang Liu[1], Congjiao Sun[1], Yiyuan Yan[12], Guangqi Li[2], Fengying Shi[2], Guiqin Wu[2], Aiqiao Liu[2], Ning Yang[1*]

[1]National Engineering Laboratory for Animal Breeding and MOA Key Laboratory of Animal Genetics and Breeding, College of Animal Science and Technology, China Agricultural University, Beijing, 100193, China

[2]Beijing Engineering Research Center of Layer, Beijing, 101206, China

Table S1. Estimation of genetic parameters for egg quality traits (at 80 weeks)

Traits	ESC80	ESI80	EST80	ESS80	AH80	YC80	HU80
**ESC80**	0.36(0.06)	-0.04(0.15)	-0.19(0.19)	-0.16(0.19)	0.07(0.18)	-0.11(0.19)	0.11(0.17)
**ESI80**	-0.02	0.33(0.06)	0.11(0.19)	0.08(0.20)	-0.33(0.17)	-0.03(0.20)	-0.29(0.17)
**EST80**	-0.05	-0.07	0.19(0.06)	0.78(0.14)	-0.44(0.22)	0.14(0.25)	-0.48(0.22)
**ESS80**	-0.11	-0.07	0.44	0.17(0.06)	-0.26(0.23)	0.22(0.25)	-0.27(0.23)
**AH80**	-0.03	-0.19	-0.08	0.00	0.21(0.06)	-0.05(0.24)	0.98(0.01)
**YC80**	-0.05	0.08	0.11	0.04	-0.03	0.17(0.06)	-0.08(0.23)
**HU80**	-0.02	-0.20	-0.06	-0.01	0.97	-0.06	0.22(0.06)

Diagonal: heritability estimation (bold is SNP-based), Upper triangle: genetic correlations, Lower triangle: phenotypic correlations. Standard error are in parenthese.
Traitsa: ESC, ESI, EST, ESS, AH, YC, HU at 80 weeks of age.

Table S2 Genome-wide significant SNPs for albumen height (AH), haugh unit (HU), eggshell color(ESC) at different age.

Traits	Chr.	rs	Position	Minor/Major allele	AF	beta	se	p_value
AH72	13	rs315953420	9068564	C/A	0.457	-0.2988679	0.0531277	2.53297E-08
AH72	13	rs15694740	9018787	T/C	0.451	-0.2971442	0.0528521	2.57972E-08
AH72	13	rs313738516	9019627	C/T	0.451	-0.2953972	0.0526926	2.82223E-08
AH72	13	rs316490290	9021822	A/G	0.451	-0.2936416	0.0528179	3.65278E-08
AH72	13	rs14994619	9011631	T/A	0.456	-0.2931694	0.0529992	4.2605E-08
AH72	13	rs14994611	9013905	T/C	0.456	-0.2931694	0.0529992	4.2605E-08
AH72	13	rs312319259	9042678	C/T	0.456	-0.2935806	0.0531104	4.35124E-08
AH72	13	rs312930344	9012652	T/C	0.45	-0.2909268	0.0527256	4.59659E-08
AH72	13	rs317069247	9014006	A/G	0.45	-0.2909268	0.0527256	4.59659E-08
AH72	13	rs315968089	9038766	A/G	0.453	-0.2905494	0.0527329	4.80053E-08
AH72	13	rs317182594	9055336	C/T	0.457	-0.2915935	0.0529945	5.00183E-08
AH72	13	rs314382017	9089446	G/A	0.451	-0.2907215	0.0528451	5.02775E-08
AH72	13	rs314938285	9210433	G/C	0.45	-0.2881122	0.0526311	5.83294E-08
AH72	13	rs314255243	9053971	T/G	0.456	-0.2902131	0.053025	5.86632E-08
AH72	13	rs14994761	9104890	G/A	0.456	-0.2902131	0.053025	5.86632E-08
AH72	13	rs15695028	9036633	A/G	0.454	-0.2886942	0.0528488	6.21106E-08
AH72	13	rs312908507	9132684	T/C	0.455	-0.2882817	0.0528744	6.57372E-08
AH72	13	rs318206977	9082325	G/A	0.451	-0.2872485	0.0527987	7.0064E-08
AH72	13	rs312967486	9014834	C/T	0.447	-0.288996	0.0531262	7.03088E-08
AH72	13	rs313104119	9057724	G/T	0.45	-0.2863004	0.0526975	7.29854E-08
AH72	13	rs314380420	9083951	C/A	0.45	-0.2863004	0.0526975	7.29854E-08
AH72	13	rs80751137	9087197	G/C	0.45	-0.2863004	0.0526975	7.29854E-08
AH72	13	rs15695230	9180131	T/C	0.459	-0.2887172	0.0531874	7.48253E-08
AH72	13	rs317436738	9117287	G/A	0.454	-0.2867895	0.0528683	7.63394E-08
AH72	13	rs314451753	9156669	C/T	0.45	-0.2852536	0.0526012	7.70213E-08
AH72	13	rs315634230	9104879	A/G	0.454	-0.2868521	0.052909	7.75803E-08
AH72	13	rs14058484	9112698	T/C	0.454	-0.2868521	0.052909	7.75803E-08
AH72	13	rs317512091	9190217	C/T	0.449	-0.2851943	0.052632	7.8834E-08
AH72	13	rs315524001	9067251	A/G	0.455	-0.2867044	0.0529764	8.17436E-08
AH72	13	rs317463178	9067949	T/A	0.455	-0.2867044	0.0529764	8.17436E-08
AH72	13	rs13819310	9073294	G/A	0.455	-0.2867044	0.0529764	8.17436E-08
AH72	13	rs316719561	9158000	C/T	0.448	-0.2842548	0.0525314	8.20926E-08
AH72	13	rs13819328	9146449	T/C	0.452	-0.2845734	0.0526263	8.37465E-08
AH72	13	rs318190156	9116231	T/C	0.448	-0.2851158	0.0527393	8.43392E-08
AH72	13	rs313241993	9054032	T/C	0.454	-0.2857752	0.0528764	8.50442E-08
AH72	13	rs315976165	9091280	G/A	0.454	-0.2857752	0.0528764	8.50442E-08
AH72	13	rs80720218	9065201	C/T	0.451	-0.2854455	0.052821	8.53062E-08
AH72	13	rs13819318	9102771	A/T	0.448	-0.2851912	0.0527812	8.5651E-08
AH72	13	rs316014412	9114970	G/A	0.448	-0.2851912	0.0527812	8.5651E-08

AH72	13	rs315708988	9122919	T/C	0.454	-0.2858365	0.0529575	8.83687E-08
AH72	13	rs313079757	9135672	A/G	0.451	-0.2837947	0.0526657	9.26828E-08
AH72	13	rs14058460	9094862	C/G	0.455	-0.2845709	0.052858	9.51634E-08
AH72	13	rs315430810	9177539	A/G	0.452	-0.2837022	0.0527069	9.56993E-08
AH72	13	rs316671214	9137821	T/G	0.472	-0.290646	0.0540383	9.78408E-08
AH72	13	rs316951527	9131212	A/G	0.453	-0.2842387	0.0528542	9.82244E-08
AH72	13	rs316247737	9060501	G/A	0.449	-0.2831162	0.0526747	9.98116E-08
AH72	13	rs317596876	9069732	T/C	0.449	-0.2831162	0.0526747	9.98116E-08
AH72	13	rs314152812	9075536	C/A	0.449	-0.2831162	0.0526747	9.98116E-08
AH72	13	rs313759352	9140817	C/T	0.447	-0.2816052	0.0525553	1.09054E-07
AH72	13	rs14994826	9187788	C/T	0.447	-0.2816052	0.0525553	1.09054E-07
AH72	13	rs317485133	9198635	G/A	0.449	-0.2830583	0.0528307	1.09302E-07
AH72	13	rs316345758	9000561	G/A	0.444	-0.2855526	0.0533695	1.13683E-07
AH72	13	rs14994789	9147087	C/G	0.448	-0.2828429	0.0529326	1.18029E-07
AH72	13	rs314231622	9235072	C/T	0.447	-0.2810331	0.0526941	1.24593E-07
AH72	13	rs316419203	9093955	C/A	0.455	-0.2822429	0.0530733	1.35166E-07
AH72	13	rs316370788	8998282	T/C	0.44	-0.2825444	0.053148	1.36464E-07
AH72	13	rs315111657	9176548	A/G	0.45	-0.2795504	0.0526844	1.43939E-07
AH72	13	rs315234432	9183798	T/C	0.45	-0.2795504	0.0526844	1.43939E-07
AH72	13	rs314880512	9217448	T/C	0.45	-0.2795504	0.0526844	1.43939E-07
AH72	13	rs312763735	9008792	G/C	0.444	-0.2819718	0.0533307	1.59088E-07
AH72	13	rs80720476	9006752	A/G	0.439	-0.2799506	0.0530647	1.69122E-07
AH72	13	rs317762029	9001892	T/C	0.443	-0.2793721	0.0532473	1.96962E-07
AH72	13	rs314473268	8985142	T/C	0.446	-0.2803982	0.0534731	2.0006E-07
AH72	13	rs315829242	9220996	C/A	0.454	-0.2752211	0.0527468	2.29166E-07
AH72	13	rs318218272	8993365	G/A	0.445	-0.2777808	0.0533887	2.47561E-07
AH72	13	rs80574565	8978179	A/T	0.438	-0.276306	0.053207	2.60727E-07
AH72	13	rs316543746	8989178	C/T	0.445	-0.2770317	0.0533527	2.61515E-07
AH72	13	rs316567567	8972155	C/G	0.439	-0.2766815	0.0532869	2.6172E-07
AH72	13	rs316096826	8972968	G/A	0.439	-0.2766815	0.0532869	2.6172E-07
AH72	13	rs15695238	9186255	T/C	0.457	-0.2750031	0.0529858	2.647E-07
AH72	13	rs313886272	8998424	T/C	0.437	-0.2753555	0.0531033	2.71454E-07
AH72	13	rs315694449	8978399	T/A	0.439	-0.2754495	0.0532271	2.86351E-07
AH72	13	rs313246295	8976743	A/C	0.447	-0.2759386	0.0533802	2.94913E-07
AH72	13	rs80586493	8967990	G/A	0.447	-0.2766658	0.0535769	3.03293E-07
AH72	13	rs315976760	8993391	A/G	0.439	-0.2737258	0.0532404	3.40827E-07
AH72	13	rs318087503	8953223	T/C	0.439	-0.2739548	0.0533098	3.45074E-07
AH72	13	rs317158920	8955473	T/A	0.439	-0.2739548	0.0533098	3.45074E-07
AH72	13	rs316930344	9313579	G/A	0.453	-0.2612523	0.051896	5.89414E-07
AH72	13	rs312397772	9314696	A/G	0.457	-0.260655	0.0520718	6.80536E-07
AH72	13	rs313242340	9299502	A/G	0.45	-0.2585445	0.0521694	8.73791E-07
AH72	13	rs316302444	9288138	G/A	0.458	-0.257242	0.0521549	9.82848E-07
AH72	13	rs315816578	9291568	T/C	0.459	-0.2570146	0.0521939	1.02294E-06
AH72	13	rs316471993	9299533	A/G	0.456	-0.2563213	0.0520956	1.04352E-06

AH72	13	rs317894407	9283752	T/C	0.454	-0.2549224	0.052051	1.1675E-06
AH72	13	rs313619844	9284234	G/A	0.454	-0.2549224	0.052051	1.1675E-06
HU72	13	rs315953420	9068564	C/A	0.457	-0.3392629	0.0521311	1.3208E-10
HU72	13	rs15694740	9018787	T/C	0.451	-0.3332887	0.0518625	2.20615E-10
HU72	13	rs313738516	9019627	C/T	0.451	-0.3312099	0.0517031	2.5062E-10
HU72	13	rs317182594	9055336	C/T	0.457	-0.3315682	0.0519708	2.94907E-10
HU72	13	rs312319259	9042678	C/T	0.456	-0.3323557	0.0521001	2.96227E-10
HU72	13	rs316490290	9021822	A/G	0.451	-0.3303829	0.0518296	3.05142E-10
HU72	13	rs312930344	9012652	T/C	0.45	-0.3286491	0.0517278	3.47604E-10
HU72	13	rs317069247	9014006	A/G	0.45	-0.3286491	0.0517278	3.47604E-10
HU72	13	rs14994619	9011631	T/A	0.456	-0.3304112	0.0520063	3.47908E-10
HU72	13	rs14994611	9013905	T/C	0.456	-0.3304112	0.0520063	3.47908E-10
HU72	13	rs314382017	9089446	G/A	0.451	-0.3292724	0.0518483	3.53566E-10
HU72	13	rs312908507	9132684	T/C	0.455	-0.3287243	0.0518902	3.89641E-10
HU72	13	rs314938285	9210433	G/C	0.45	-0.3270233	0.0516544	3.99425E-10
HU72	13	rs314255243	9053971	T/G	0.456	-0.3292742	0.0520237	4.03573E-10
HU72	13	rs14994761	9104890	G/A	0.456	-0.3292742	0.0520237	4.03573E-10
HU72	13	rs315968089	9038766	A/G	0.453	-0.3272672	0.0517372	4.13003E-10
HU72	13	rs15695230	9180131	T/C	0.459	-0.3294983	0.0521994	4.48273E-10
HU72	13	rs15695028	9036633	A/G	0.454	-0.3271466	0.0518456	4.54627E-10
HU72	13	rs313104119	9057724	G/T	0.45	-0.3260448	0.0516904	4.61323E-10
HU72	13	rs314380420	9083951	C/A	0.45	-0.3260448	0.0516904	4.61323E-10
HU72	13	rs80751137	9087197	G/C	0.45	-0.3260448	0.0516904	4.61323E-10
HU72	13	rs314451753	9156669	C/T	0.45	-0.3250319	0.0516214	4.94194E-10
HU72	13	rs318206977	9082325	G/A	0.451	-0.3261617	0.0518031	4.95057E-10
HU72	13	rs316719561	9158000	C/T	0.448	-0.3244041	0.0515424	5.0196E-10
HU72	13	rs313241993	9054032	T/C	0.454	-0.3260353	0.0518647	5.26178E-10
HU72	13	rs315976165	9091280	G/A	0.454	-0.3260353	0.0518647	5.26178E-10
HU72	13	rs316951527	9131212	A/G	0.453	-0.3259019	0.0518498	5.2863E-10
HU72	13	rs317512091	9190217	C/T	0.449	-0.3245276	0.0516546	5.38002E-10
HU72	13	rs315524001	9067251	A/G	0.455	-0.3261287	0.0519763	5.65356E-10
HU72	13	rs317463178	9067949	T/A	0.455	-0.3261287	0.0519763	5.65356E-10
HU72	13	rs13819310	9073294	G/A	0.455	-0.3261287	0.0519763	5.65356E-10
HU72	13	rs13819318	9102771	A/T	0.448	-0.3247835	0.0517757	5.71165E-10
HU72	13	rs316014412	9114970	G/A	0.448	-0.3247835	0.0517757	5.71165E-10
HU72	13	rs315430810	9177539	A/G	0.452	-0.3243639	0.0517135	5.73158E-10
HU72	13	rs13819328	9146449	T/C	0.452	-0.3239547	0.0516494	5.7367E-10
HU72	13	rs315634230	9104879	A/G	0.454	-0.3254768	0.0519173	5.84463E-10
HU72	13	rs14058484	9112698	T/C	0.454	-0.3254768	0.0519173	5.84463E-10
HU72	13	rs80720218	9065201	C/T	0.451	-0.324618	0.0518243	6.03842E-10
HU72	13	rs318190156	9116231	T/C	0.448	-0.3240527	0.0517436	6.08155E-10
HU72	13	rs317436738	9117287	G/A	0.454	-0.324762	0.0518864	6.21546E-10
HU72	13	rs316247737	9060501	G/A	0.449	-0.3232512	0.0516681	6.3227E-10
HU72	13	rs317596876	9069732	T/C	0.449	-0.3232512	0.0516681	6.3227E-10

HU72	13	rs314152812	9075536	C/A	0.449	-0.3232512	0.0516681	6.3227E-10
HU72	13	rs315708988	9122919	T/C	0.454	-0.3250179	0.0519782	6.45309E-10
HU72	13	rs313079757	9135672	A/G	0.451	-0.3230977	0.0516986	6.58575E-10
HU72	13	rs313759352	9140817	C/T	0.447	-0.3222083	0.0515648	6.62722E-10
HU72	13	rs14994826	9187788	C/T	0.447	-0.3222083	0.0515648	6.62722E-10
HU72	13	rs14058460	9094862	C/G	0.455	-0.3236291	0.0518707	7.02142E-10
HU72	13	rs317485133	9198635	G/A	0.449	-0.3231165	0.0518515	7.35359E-10
HU72	13	rs316671214	9137821	T/G	0.472	-0.3295649	0.0530492	8.26245E-10
HU72	13	rs315111657	9176548	A/G	0.45	-0.3208262	0.0516884	8.5439E-10
HU72	13	rs315234432	9183798	T/C	0.45	-0.3208262	0.0516884	8.5439E-10
HU72	13	rs314880512	9217448	T/C	0.45	-0.3208262	0.0516884	8.5439E-10
HU72	13	rs314231622	9235072	C/T	0.447	-0.3202431	0.0517258	9.40157E-10
HU72	13	rs14994789	9147087	C/G	0.448	-0.3215615	0.0519521	9.49293E-10
HU72	13	rs316419203	9093955	C/A	0.455	-0.3217544	0.0520808	1.01836E-09
HU72	13	rs312967486	9014834	C/T	0.447	-0.3222252	0.052171	1.02861E-09
HU72	13	rs15695238	9186255	T/C	0.457	-0.3172615	0.0520135	1.63233E-09
HU72	13	rs315829242	9220996	C/A	0.454	-0.3148708	0.0517845	1.83074E-09
HU72	13	rs316345758	9000561	G/A	0.444	-0.3163769	0.052482	2.49592E-09
HU72	13	rs316370788	8998282	T/C	0.44	-0.3135076	0.0522657	2.97946E-09
HU72	13	rs80720476	9006752	A/G	0.439	-0.3119073	0.0521726	3.35122E-09
HU72	13	rs312763735	9008792	G/C	0.444	-0.3133944	0.0524447	3.40428E-09
HU72	13	rs317762029	9001892	T/C	0.443	-0.3117932	0.0523513	3.82676E-09
HU72	13	rs316930344	9313579	G/A	0.453	-0.3022323	0.0509397	4.3693E-09
HU72	13	rs314473268	8985142	T/C	0.446	-0.3119249	0.0525962	4.43549E-09
HU72	13	rs312397772	9314696	A/G	0.457	-0.3021482	0.0511109	4.95378E-09
HU72	13	rs318218272	8993365	G/A	0.445	-0.3103013	0.0525016	4.99138E-09
HU72	13	rs313242340	9299502	A/G	0.45	-0.3022588	0.0511862	5.1458E-09
HU72	13	rs316567567	8972155	C/G	0.439	-0.3091295	0.0524021	5.32573E-09
HU72	13	rs316096826	8972968	G/A	0.439	-0.3091295	0.0524021	5.32573E-09
HU72	13	rs313246295	8976743	A/C	0.447	-0.3095845	0.0524803	5.32913E-09
HU72	13	rs313886272	8998424	T/C	0.437	-0.3075111	0.0522134	5.63339E-09
HU72	13	rs80574565	8978179	A/T	0.438	-0.307857	0.0523272	5.83988E-09
HU72	13	rs315694449	8978399	T/A	0.439	-0.3075547	0.0523441	6.10581E-09
HU72	13	rs316543746	8989178	C/T	0.445	-0.3081409	0.052484	6.26649E-09
HU72	13	rs316471993	9299533	A/G	0.456	-0.2999381	0.0511206	6.40891E-09
HU72	13	rs80586493	8967990	G/A	0.447	-0.3089861	0.0527061	6.59058E-09
HU72	13	rs318087503	8953223	T/C	0.439	-0.3068632	0.0524234	6.93769E-09
HU72	13	rs317158920	8955473	T/A	0.439	-0.3068632	0.0524234	6.93769E-09
HU72	13	rs316302444	9288138	G/A	0.458	-0.2996115	0.0511904	6.96418E-09
HU72	13	rs317894407	9283752	T/C	0.454	-0.2986861	0.0510753	7.16553E-09
HU72	13	rs313619844	9284234	G/A	0.454	-0.2986861	0.0510753	7.16553E-09
HU72	13	rs315816578	9291568	T/C	0.459	-0.2994124	0.0512355	7.33726E-09
HU72	13	rs315976760	8993391	A/G	0.439	-0.3059727	0.0523592	7.34272E-09
HU80	13	rs15695238	9186255	T/C	0.455	-0.2792849	0.0562737	8.62139E-07

HU80	13	rs14994789	9147087	C/G	0.442	-0.2795498	0.0564306	9.02154E-07
HU80	13	rs318206977	9082325	G/A	0.446	-0.2770068	0.0562113	1.02602E-06
HU80	13	rs15694740	9018787	T/C	0.446	-0.2770185	0.0562537	1.04397E-06
HU80	13	rs316419203	9093955	C/A	0.453	-0.278429	0.0565671	1.05618E-06
HU80	13	rs80720218	9065201	C/T	0.447	-0.2765477	0.0562546	1.08853E-06
HU80	13	rs317512091	9190217	C/T	0.444	-0.2743087	0.0559622	1.16831E-06
HU80	13	rs316247737	9060501	G/A	0.444	-0.2748653	0.0561315	1.19667E-06
HU80	13	rs317596876	9069732	T/C	0.444	-0.2748653	0.0561315	1.19667E-06
HU80	13	rs314152812	9075536	C/A	0.444	-0.2748653	0.0561315	1.19667E-06
HU80	13	rs312930344	9012652	T/C	0.446	-0.2748885	0.0561371	1.19712E-06
HU80	13	rs317069247	9014006	A/G	0.446	-0.2748885	0.0561371	1.19712E-06
HU80	13	rs15695230	9180131	T/C	0.457	-0.2770171	0.0566443	1.23463E-06
HU80	13	rs315968089	9038766	A/G	0.448	-0.2739514	0.0560878	1.27246E-06
HU80	13	rs313759352	9140817	C/T	0.442	-0.2726156	0.0559252	1.33444E-06
HU80	13	rs315524001	9067251	A/G	0.453	-0.2749317	0.0564768	1.37837E-06
HU80	13	rs317463178	9067949	T/A	0.453	-0.2749317	0.0564768	1.37837E-06
HU80	13	rs13819310	9073294	G/A	0.453	-0.2749317	0.0564768	1.37837E-06
HU80	13	rs316490290	9021822	A/G	0.447	-0.2732759	0.056227	1.43217E-06
HU80	13	rs317485133	9198635	G/A	0.444	-0.2732078	0.0562335	1.44469E-06
HU80	13	rs314382017	9089446	G/A	0.448	-0.2737292	0.0563496	1.45004E-06
ESC72	12	rs315306430	2849211	T/C	0.454	-0.2822241	0.0528912	1.21676E-07
ESC72	12	rs316672749	2844732	C/T	0.446	-0.2726492	0.0526942	2.85101E-07
ESC72	12	rs315932763	2803865	C/T	0.483	-0.2701584	0.0522891	2.96498E-07
ESC72	12	rs14032349	2782392	C/T	0.488	-0.2653294	0.0516412	3.43928E-07
ESC72	12	rs10723545	2797513	C/T	0.483	-0.2681299	0.0522159	3.49144E-07
ESC72	12	rs314576112	2813468	G/A	0.466	-0.2696797	0.0525702	3.58496E-07
ESC72	12	rs14971525	2673944	G/A	0.461	-0.2658029	0.051915	3.77323E-07
ESC72	12	rs14032300	2746354	G/T	0.493	-0.2632568	0.052338	5.97473E-07
ESC72	12	rs314358128	2806403	C/G	0.476	-0.2609071	0.0521952	6.99679E-07
ESC72	12	rs317860570	2912769	C/T	0.397	-0.2688175	0.0537839	7.01709E-07
ESC72	12	rs317268129	2931515	G/A	0.365	-0.2757663	0.0553813	7.70905E-07
ESC72	12	rs14032285	2720852	G/A	0.492	-0.2594158	0.0523162	8.55608E-07
ESC72	12	rs315593584	2710317	T/C	0.49	-0.2596441	0.052537	9.29052E-07
ESC72	12	rs317175667	2819841	C/T	0.456	-0.2559327	0.0523508	1.21061E-06
ESC72	12	rs13621598	2898833	T/C	0.383	-0.2602894	0.0533257	1.25738E-06
ESC72	12	rs314478643	2711829	T/C	0.486	-0.2539994	0.0522398	1.38041E-06
ESC72	12	rs315895174	2716771	T/C	0.486	-0.2539994	0.0522398	1.38041E-06
ESC72	12	rs14971630	2724110	C/G	0.486	-0.2539994	0.0522398	1.38041E-06
ESC72	12	rs15633640	2751117	T/C	0.486	-0.2539994	0.0522398	1.38041E-06
ESC72	12	rs15633657	2752107	G/A	0.486	-0.2539994	0.0522398	1.38041E-06
ESC72	12	rs315825335	2757654	G/A	0.486	-0.2539994	0.0522398	1.38041E-06
ESC72	12	rs314164115	2758853	T/C	0.486	-0.2539994	0.0522398	1.38041E-06
ESC72	12	rs315673085	2658337	C/T	0.358	0.2710872	0.055838	1.43078E-06
ESC72	12	rs15633943	2891561	C/T	0.39	-0.2589244	0.053478	1.52641E-06

ESC72	21	rs315046589	2588765	C/T	0.443	-0.2709245	0.0527974	3.55728E-07
ESC72	21	rs15182085	2576040	T/C	0.449	-0.2724541	0.0531714	3.6942E-07
ESC72	21	rs314955180	2567490	T/C	0.432	-0.2714208	0.0531383	4.01617E-07
ESC72	21	rs317521562	2551092	A/G	0.353	-0.276146	0.0544271	4.78306E-07
ESC72	21	rs14283106	2546399	G/A	0.352	-0.2756204	0.0543503	4.8445E-07
ESC72	21	rs16179050	2547410	T/A	0.435	-0.2675696	0.0528508	5.05785E-07
ESC72	21	rs13603074	2576858	T/C	0.357	-0.2792286	0.0552815	5.36817E-07
ESC72	21	rs312991901	2581820	C/T	0.446	-0.2651194	0.0524902	5.3736E-07
ESC72	21	rs317565991	2577321	T/C	0.347	-0.2756355	0.0545807	5.3949E-07
ESC72	21	rs316467747	2581177	C/T	0.442	-0.2663172	0.0527569	5.45135E-07
ESC72	21	rs313596544	2530096	A/G	0.432	-0.2683681	0.0531763	5.48609E-07
ESC72	21	rs316368024	2530993	G/A	0.432	-0.2683681	0.0531763	5.48609E-07
ESC72	21	rs13603073	2571403	G/A	0.438	-0.267442	0.0530418	5.61739E-07
ESC72	21	rs317148194	2549255	C/T	0.436	-0.2656555	0.0528505	6.07829E-07
ESC72	21	rs16179061	2553019	T/C	0.436	-0.2656555	0.0528505	6.07829E-07
ESC72	21	rs16179062	2553043	C/T	0.436	-0.2656555	0.0528505	6.07829E-07
ESC72	21	rs316938127	2553710	A/G	0.436	-0.2656555	0.0528505	6.07829E-07
ESC72	21	rs313522691	2589799	A/G	0.356	-0.2720563	0.0541658	6.19898E-07
ESC72	21	rs313283638	2579464	G/A	0.359	-0.2708906	0.0539672	6.2975E-07
ESC72	21	rs318218414	2585852	T/C	0.359	-0.2708906	0.0539672	6.2975E-07
ESC72	21	rs16179081	2575378	A/G	0.35	-0.2712356	0.0542867	7.07919E-07
ESC72	21	rs315518467	2522760	T/G	0.432	-0.2646044	0.0531972	7.91993E-07
ESC72	21	rs315602443	2528083	T/C	0.435	-0.2625976	0.0528652	8.19173E-07
ESC72	21	rs313388623	2584552	A/G	0.357	-0.2682876	0.0541617	8.77922E-07
ESC72	21	rs312985859	2563984	G/A	0.351	-0.2701655	0.0545571	8.84468E-07
ESC72	21	rs14283124	2564009	T/C	0.351	-0.2701655	0.0545571	8.84468E-07
ESC72	21	rs14283126	2565424	G/A	0.351	-0.2701655	0.0545571	8.84468E-07
ESC72	21	rs313340132	2574139	C/T	0.351	-0.2701655	0.0545571	8.84468E-07
ESC72	21	rs313368631	2574259	T/C	0.351	-0.2701655	0.0545571	8.84468E-07
ESC72	21	rs315009435	2552480	T/G	0.436	-0.2608338	0.0527699	9.25671E-07
ESC72	21	rs317711513	2521601	C/T	0.432	-0.2607672	0.0531514	1.11121E-06
ESC72	21	rs313390206	2559470	G/A	0.433	-0.2607915	0.0531717	1.11901E-06
ESC72	21	rs316480863	2556029	A/C	0.435	-0.25983	0.0529928	1.12782E-06
ESC72	21	rs312725518	2568887	C/T	0.435	-0.25983	0.0529928	1.12782E-06
ESC72	21	rs14283151	2589483	G/T	0.457	-0.2608612	0.0534421	0.000001257
ESC72	21	rs312993354	2529041	C/A	0.434	-0.2597071	0.0533451	1.33859E-06
ESC72	21	rs313841898	2560219	A/G	0.434	-0.2569795	0.0531261	1.56067E-06
ESC80	21	rs14283106	2546399	G/A	0.356	-0.2974156	0.0574674	2.92E-07
ESC80	21	rs317521562	2551092	A/G	0.357	-0.2908837	0.0575684	5.46E-07
ESC80	21	rs312985859	2563984	G/A	0.354	-0.2908104	0.0577986	6.09E-07
ESC80	21	rs14283124	2564009	T/C	0.354	-0.2908104	0.0577986	6.09E-07
ESC80	21	rs14283126	2565424	G/A	0.354	-0.2908104	0.0577986	6.09E-07
ESC80	21	rs313340132	2574139	C/T	0.354	-0.2908104	0.0577986	6.09E-07
ESC80	21	rs313368631	2574259	T/C	0.354	-0.2908104	0.0577986	6.09E-07

ESC80	21	rs13603074	2576858	T/C	0.361	-0.2930354	0.0589205	8.15E-07
ESC80	21	rs16179081	2575378	A/G	0.353	-0.285171	0.0575227	8.82E-07

Figure S1 Manhattan plots and Q-Q plots of genome-wide association study for egg quality traits.

A

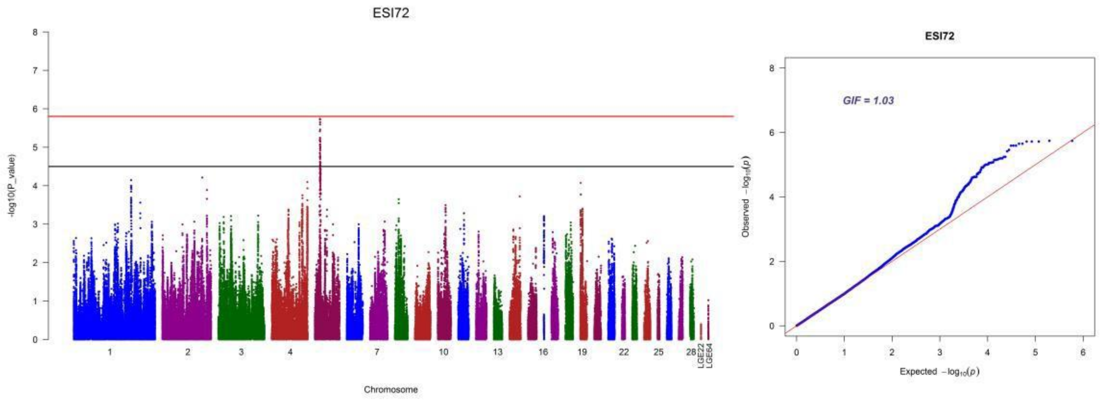

B

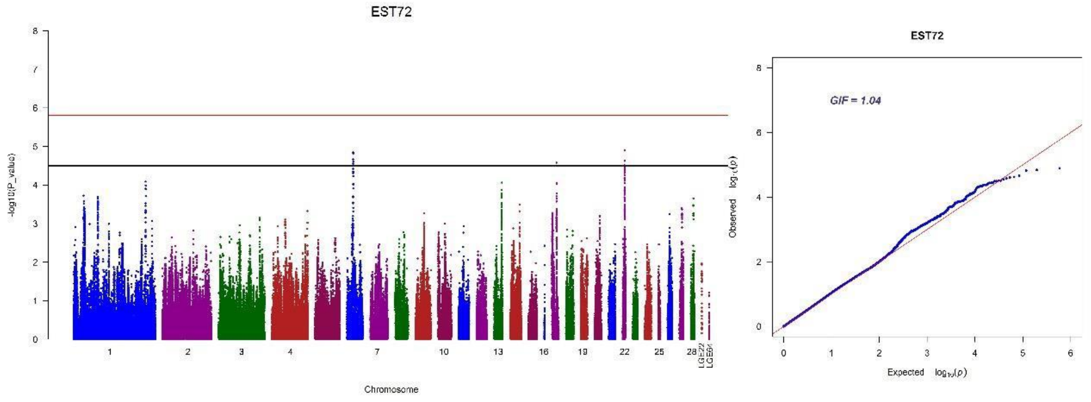

C

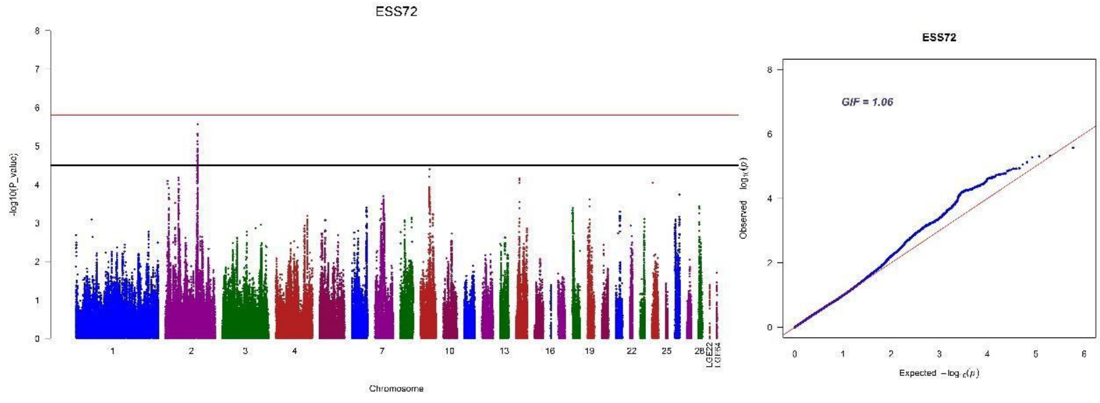

D

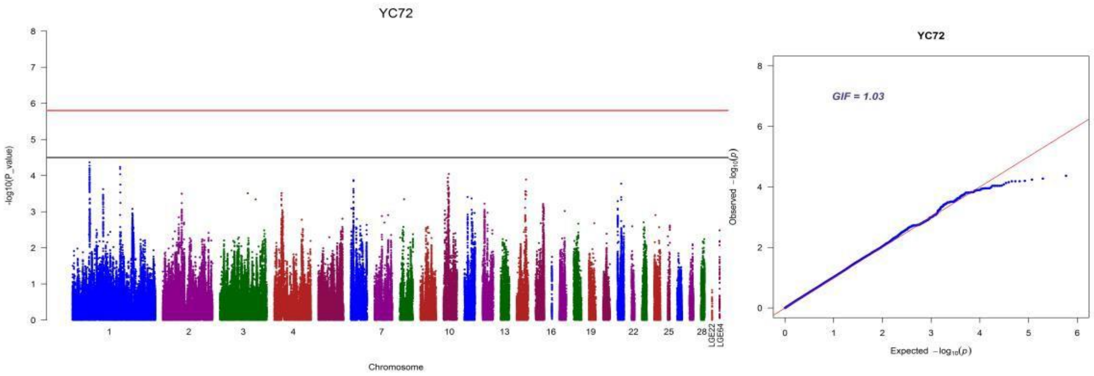
YC72
-log10(P_value)
Chromosome
Observed −log10(p)
Expected −log10(p)
GIF = 1.03

E

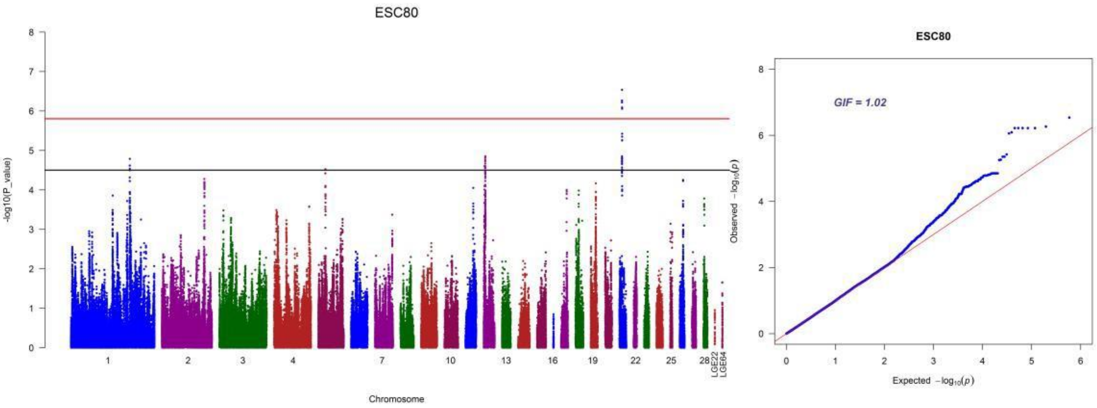
ESC80
-log10(P_value)
Chromosome
Observed −log10(p)
Expected −log10(p)
GIF = 1.02

F

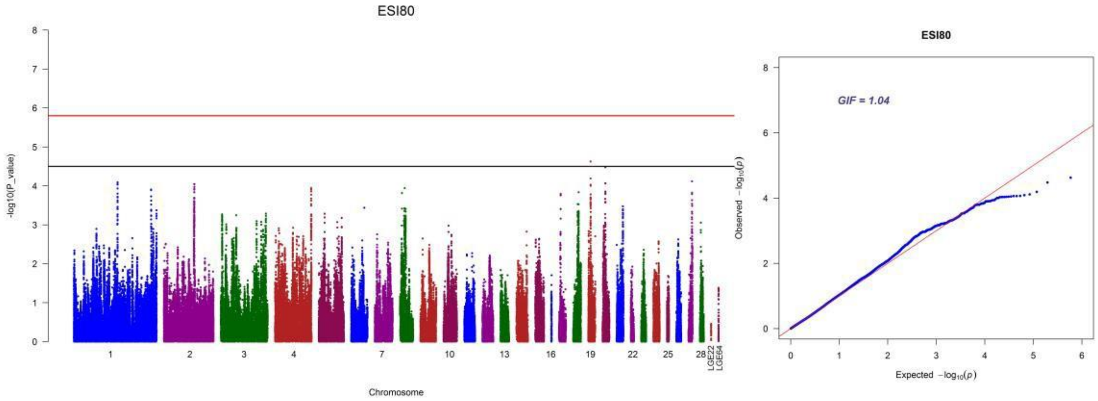
ESI80
-log10(P_value)
Chromosome
Observed −log10(p)
Expected −log10(p)
GIF = 1.04

G

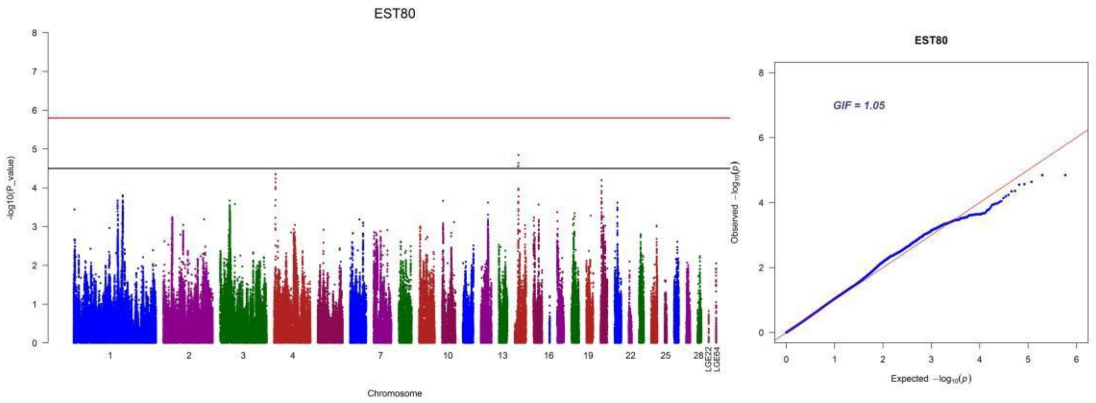
EST80
-log10(P_value)
Chromosome
EST80
GIF = 1.05
Observed −log10(p)
Expected −log10(p)

H

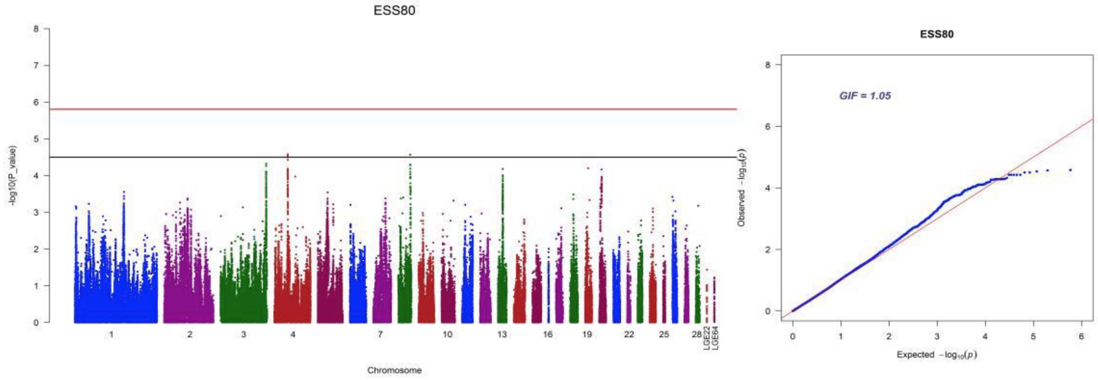
ESS80
-log10(P_value)
Chromosome
ESS80
GIF = 1.05
Observed −log10(p)
Expected −log10(p)

I

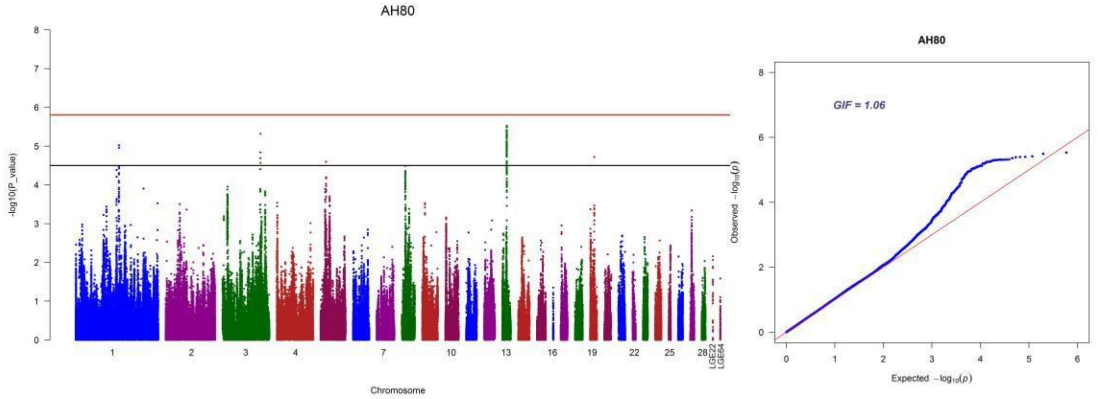
AH80
-log10(P_value)
Chromosome
AH80
GIF = 1.06
Observed −log10(p)
Expected −log10(p)

J

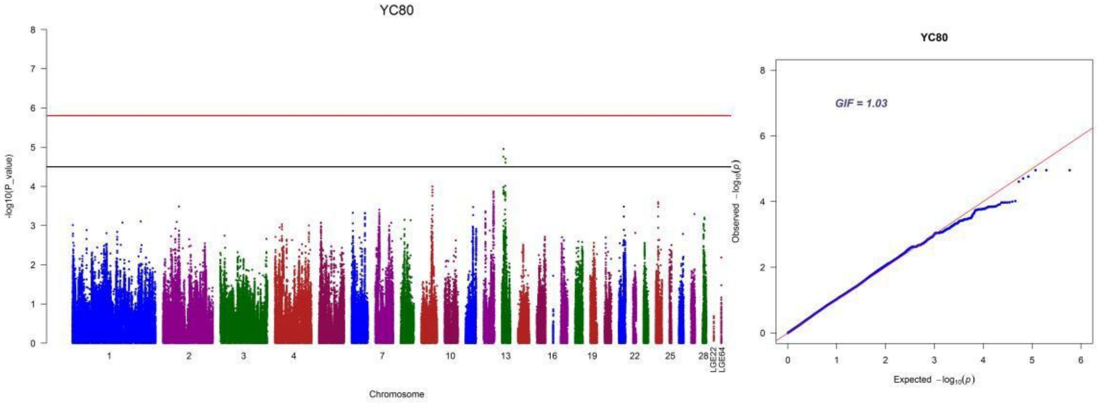
YC80
-log10(P_value)
Chromosome
YC80
GIF = 1.03
Observed −log10(p)
Expected −log10(p)

K

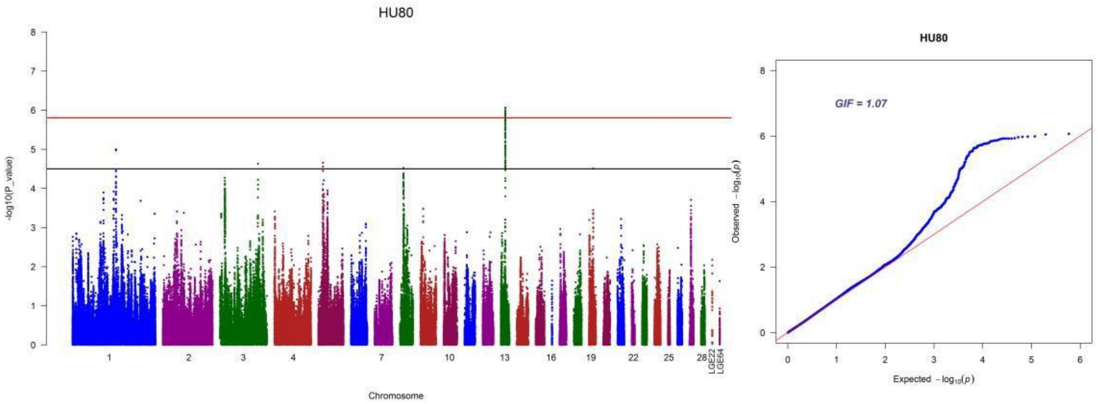
HU80
-log10(P_value)
Chromosome
1 2 3 4 7 10 13 16 19 22 25 28 LGE22 LGE64
HU80
GIF = 1.07
Observed −log10(p)
Expected −log10(p)

L

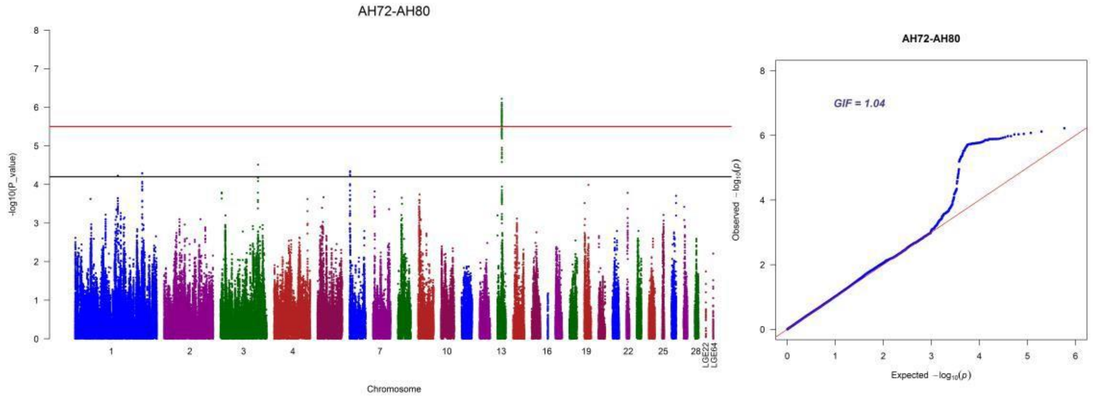
AH72-AH80
-log10(P_value)
Chromosome
1 2 3 4 7 10 13 16 19 22 25 28 LGE22 LGE64
AH72-AH80
GIF = 1.04
Observed −log10(p)
Expected −log10(p)

M

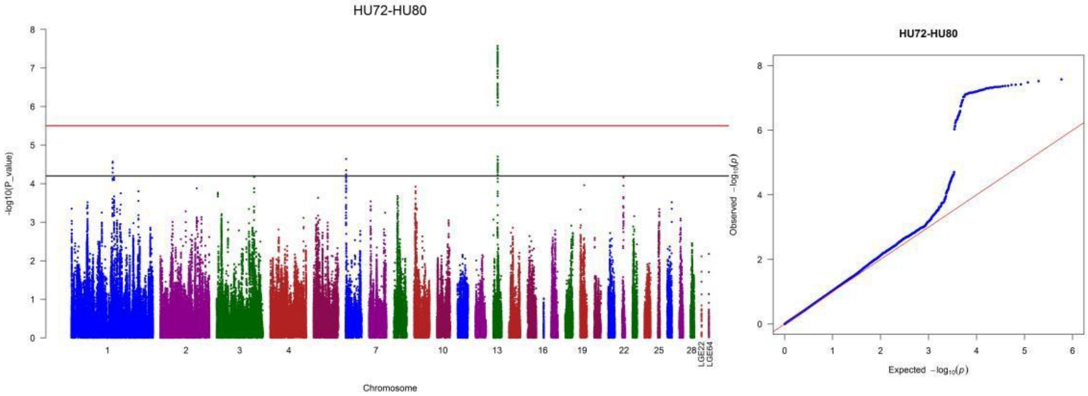
HU72-HU80
-log10(P_value)
Chromosome
1 2 3 4 7 10 13 16 19 22 25 28 LGE22 LGE64
HU72-HU80
Observed −log10(p)
Expected −log10(p)

N

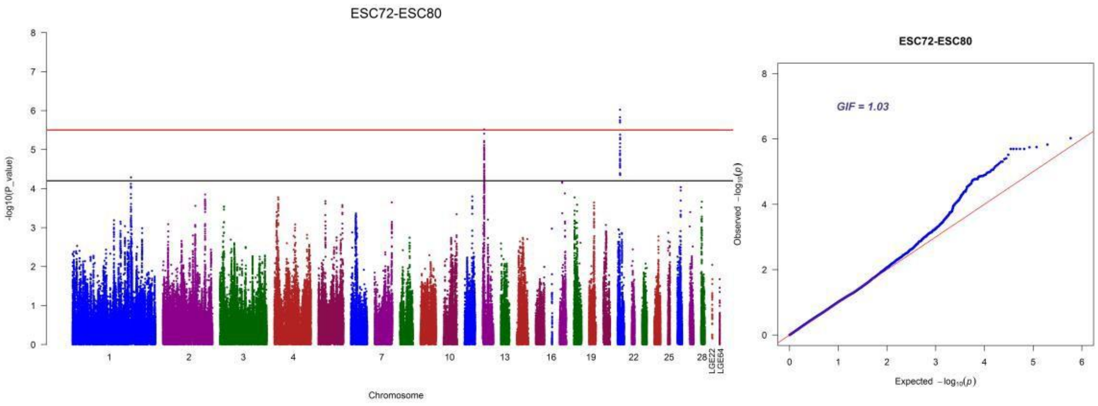
ESC72-ESC80
-log10(P_value)
Chromosome
1
2
3
4
7
10
13
16
19
22
25
28
LGE22
LGE64
ESC72-ESC80
GIF = 1.03
Observed −log10(p)
Expected −log10(p)

Figure S2 LD analysis of loci in the significant region.

A: Linkage disequilibrium (LD) analysis of significant SNPs at HU72

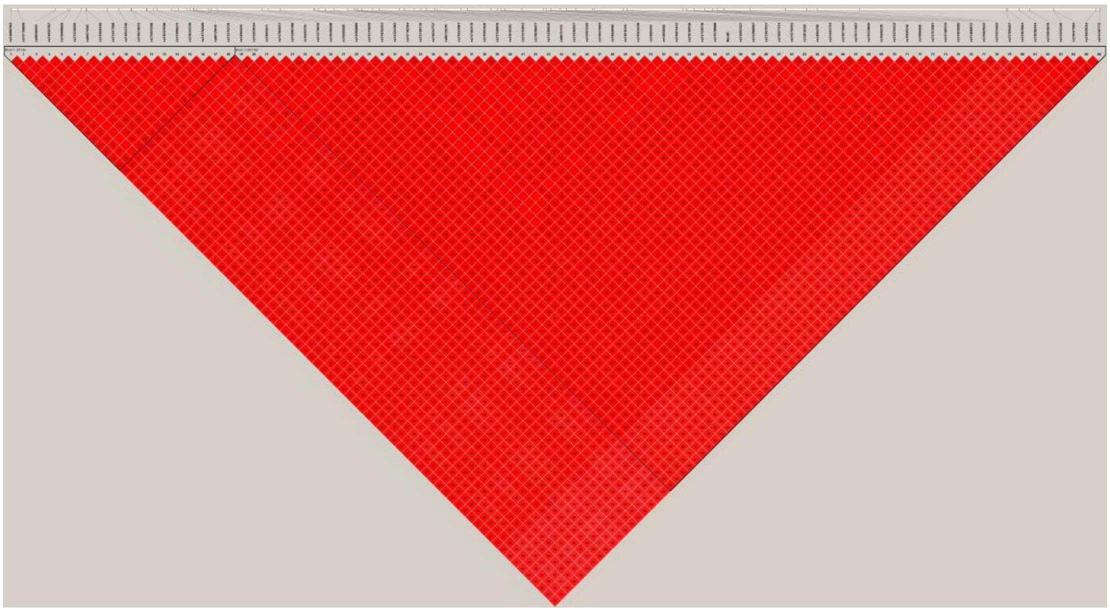

B: Linkage disequilibrium (LD) analysis of significant SNPs at HU80

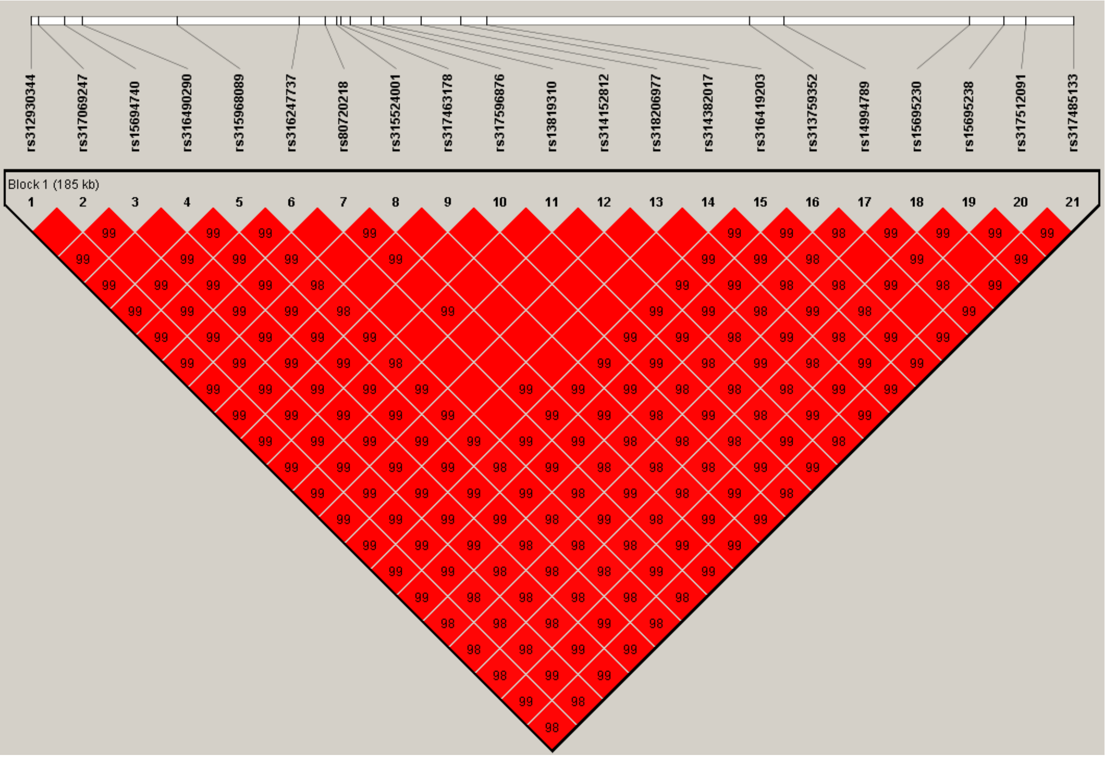

C: Linkage disequilibrium (LD) analysis of significant SNPs on CHR12 at ESC72

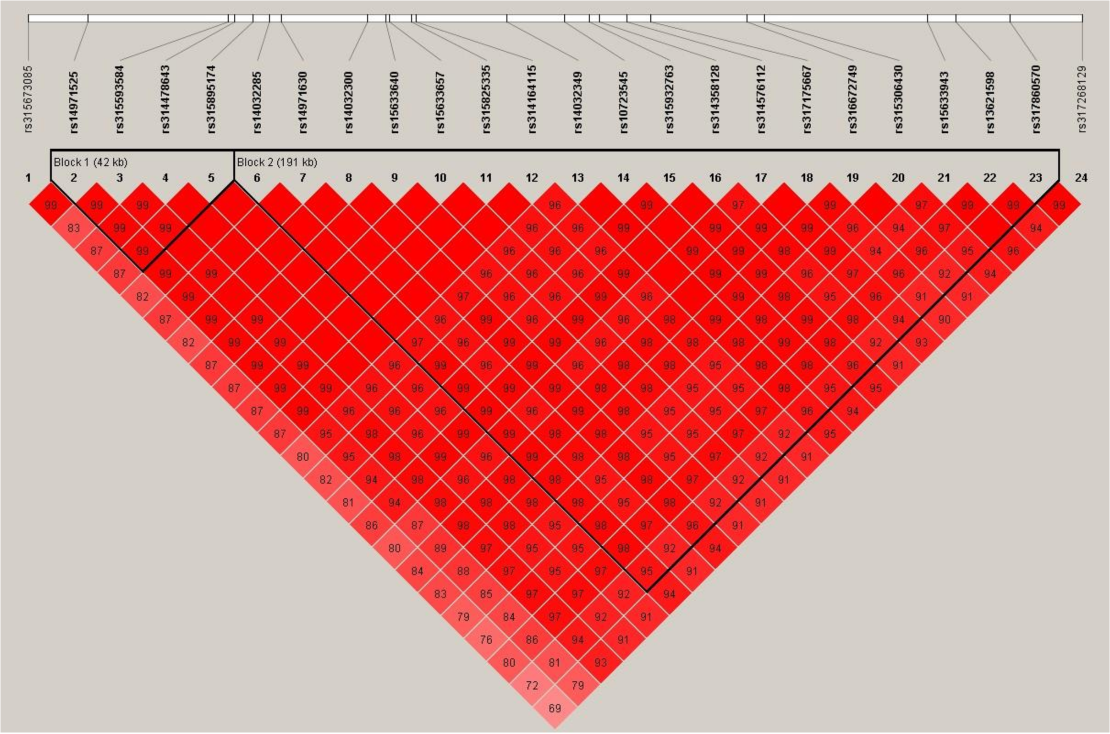

D: Linkage disequilibrium (LD) analysis of significant SNPs on CHR21 at ESC72

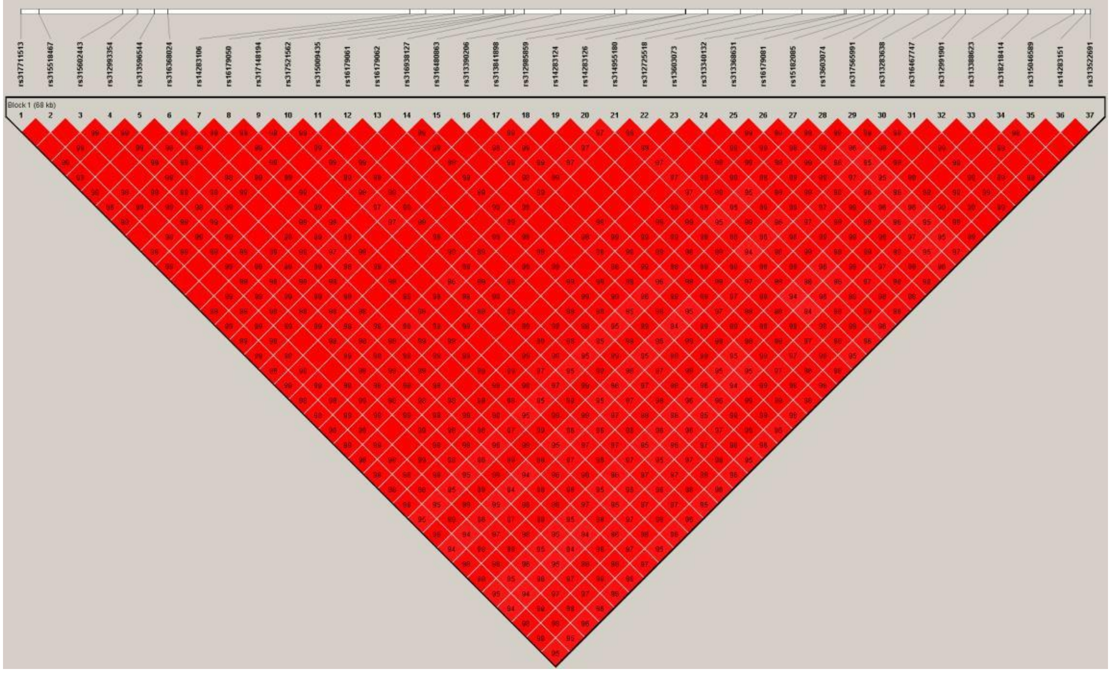

E: Linkage disequilibrium (LD) analysis of significant SNPs at ESC80

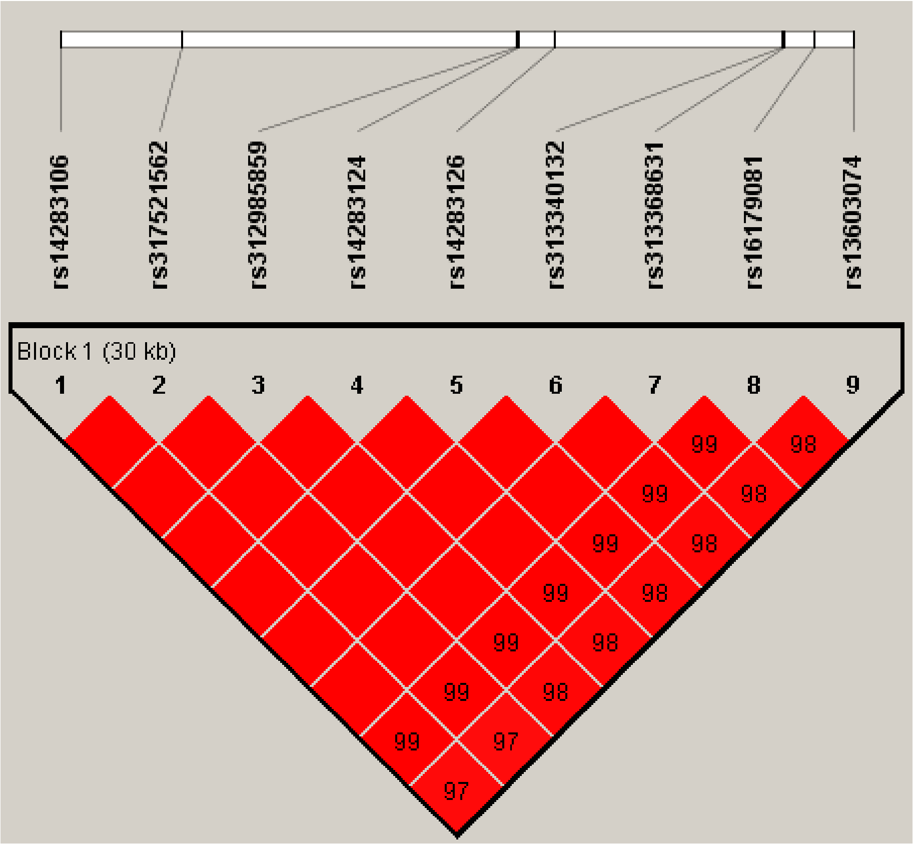
rs14283106
rs317521562
rs312985859
rs14283124
rs14283126
rs313340132
rs313368631
rs16179081
rs13603074
Block 1 (30 kb)
1
2
3
4
5
6
7
8
9
99
98
99
98
99
98
99
98
99
98
99
98
99
97
97
